# Social distancing and changes in drug use: Results from a cross-sectional study during the COVID-19 pandemic in Brazil

**DOI:** 10.3389/fpsyt.2022.999372

**Published:** 2022-11-09

**Authors:** Maurício Schüler Nin, Nubia Heidrich, Felipe B. Almeida, Lucas R. Izolan, Hilda M. R. M. Constant, Luana Freese, Rosane Gomez, Helena M. T. Barros

**Affiliations:** ^1^Institute of Basic Sciences, Universidade Federal do Rio Grande, Rio Grande, Brazil; ^2^Graduate Program in Health Sciences, Universidade Federal de Ciências da Saúde de Porto Alegre, Porto Alegre, Brazil; ^3^Graduate Program in Neurosciences, Universidade Federal do Rio Grande do Sul, Porto Alegre, Brazil; ^4^Graduate Program in Therapeutics and Pharmacology, Universidade Federal do Rio Grande do Sul, Porto Alegre, Brazil; ^5^Department of Pharmacosciences, Universidade Federal de Ciências da Saúde de Porto Alegre, Porto Alegre, Brazil

**Keywords:** substance-related disorders, alcohol, cannabis, tobacco, social isolation, depression, anxiety, SARS-CoV-2 infection

## Abstract

**Background:**

The outbreak of coronavirus disease 19 has led to measures of social distancing and quarantine worldwide. This stressful period may lead to psychological problems, including changes in substance use. In addition, sociodemographic factors are linked to changed levels of drug use and abuse observed during the COVID-19 pandemic, which are also associated with increased anxiety, depression, and other disorders. Thus, the aim of the study was to investigate (i) changes in drug use during the COVID-19 pandemic associated with social distancing, and (ii) to verify factors associated with those changes.

**Methods:**

A web-based cross-sectional observational survey was completed by a self-selected adult general population in Brazil (*N* = 2,435) during September/October 2020 (first wave) before and throughout the pandemic. Key outcomes: social distancing, self-reported drug use (ASSIST), and emotional states (DASS-21).

**Results:**

High social distancing was associated with fewer chances (prevalence ratio) of increased drug use for alcohol (0.71, CI_95%_: 0.64–0.80), tobacco (0.72; CI_95%_: 0.60–0.87), cannabis (0.65; CI_95%_: 0.55–0.78), and others. Low social distancing presented a higher DASS-21 score for anxiety (*P* = 0.017). Concerning covariates analysis by a general linear model, men (alcohol: 1. 71; cannabis: 3.86), younger age (alcohol: 0.97), less education (alcohol, tobacco, cannabis and cocaine/crack comparing several lower schooling categories vs. higher education), lower income (alcohol: 0.42; tobacco: 0.47; and cannabis: 0.36), and higher depression DASS-21 score (alcohol: 1.05; tobacco: 1.08; cannabis: 1.07; and cocaine/crack: 1.07) were associated with higher use prevalence of several drugs.

**Conclusions:**

Individuals reporting low social distancing increased the use of most drugs during the pandemic, while high social distancing significantly decreased drug use. Anxiety and depressive states and several sociodemographic factors (men; lower income; less education) were associated with higher drug use patterns.

## Introduction

The current COVID-19 pandemic, near its end, has had several consequences in many countries, resulting from a long period of social distancing and changes in lifestyle and socioeconomic status, leading to an impact on mental health and drug use. From November 2019, when it first appeared in Wuhan, China, to September 2020, nearly one million people died from COVID-19 worldwide (1). In Brazil, the first case was detected in February 2020, and by September 2020, nearly 4 million accumulated cases were reported and approximately 13% of the world deaths were confirmed in Brazil (2). The stress caused by fear of getting infected or the risk of contaminating others increased the prevalence of anxiety, depression, and other related mental disorders (3, 4). Social distancing is also a steering element for mental health disturbance, leading to feelings of loneliness, distress, insomnia, and even anger (3, 5). Additionally, the economic consequences of social distancing regulations and the unavailability of a vaccine during the first year of the pandemic comprised factors that contributed to mental health issues (6, 7). All of these factors might be linked to changed levels of drug use and abuse observed during that same period, which is also associated with increased anxiety, depression, and stress (8, 9).

Surprisingly, studies have shown no change, increases (10–12) and decreases (9, 13–15) in drug use during the pandemic, varying according to the different types of drugs studied and the demographic characteristics of users. For instance, the frequency of alcohol consumption during the first wave decreased in Albania, Finland, Norway and Spain. However, a frequency increase was observed in Germany and the United Kingdom, while no changes were seen in several countries (10). Sociodemographic factors also have an impact on drug use patterns, with higher income, job loss, stress and depression being associated with an increase in alcohol drinking (11). Concerning social distancing and isolation, people who experienced two lockdowns also reported more frequent alcohol and cannabis consumption, compared to those who experienced only one lockdown, but no changes were observed for other illegal drugs (12). On the other hand, drugs such as ecstasy and MDMA had a reduced use after restrictions associated to social interactions during the pandemic (14). Another influencing factor, this one related to psychological aspects, is that individuals describing to be worried about getting COVID-19 presented association with higher heavy alcohol drinking (15).

Increased drug use is not only a problem, as it may also increase the chances of developing a more severe infection by COVID-19 (16, 17) or increase hospitalization risk (18). Psychostimulants, such as cocaine and methamphetamine, have shown increased overdose deaths in 2020 (19). Furthermore, an increase in marijuana use was observed in several countries, such as in the US (43.9%) (20), Canada (20%) associated with self-distancing Self-isolation: A significant contributor to cannabis use during the COVID-19 pandemic (21), and Europe (12.2%) (10).

Notably, sociodemographic characteristics are associated with drug use/abuse during the COVID-19 pandemic. Young adults seem to present higher levels of alcohol and drug abuse than older individuals (22). A nationwide survey carried out during the beginning of the pandemic found that job loss, overeating, changes in sleep, stress, and depression were associated with changes in alcohol consumption (11). Beyond that, stress symptoms related to COVID-19 infection and the tendency to disregard social distancing were both linked to substance abuse (22). Additionally, among the self-isolated individuals, 26% reported increased drug consumption as a way to deal with distancing. Indeed, stress symptoms related to COVID-19 and disregard for social distancing present association with substance use (22). Therefore, we aimed to test the hypothesis that (i) social distancing is associated with drug use changes during the COVID-19 pandemic and that (ii) self-reported drug consumption changes are associated with mental health issues and sociodemographic factors among the adult population in Brazil through an observational study using an online survey.

## Materials and methods

This Study Fully Complies with the STrengthening the Reporting of OBservational Studies in Epidemiology (STROBE) Statement Reports of Cross-Sectional Studies (23) (see Supplementary Table S1). The project, including the main hypothesis and the analysis plan, was preregistered in a national platform (PlataformaBrasil) with Protocol Number #4.165.998.

### Design

This study is a cross-sectional survey of the general population of Brazil carried out by self-responses to an online survey performed from September 9th to October 16th, 2020. The study was approved by the National Ethics Committee and Ethics Committee of Research of the Federal University of Health Sciences of Porto Alegre (#4241378).

### Participants

The sample was achieved through sharing of the survey link after the peak of the first COVID-19 wave, lasting almost to the end of Brazil's first wave of the pandemic. Potential subjects were reached through WhatsApp texting app, emails provided by the university, social media paid ads and boosting on Facebook^®^ and Instagram^®^ (no filters used), as well as through ads posted on universities' social media pages. Potential “clickers” would see an ad with the survey's poster, with the main question: “How is your use of alcohol and other drugs during the COVID-19 pandemic?” on a link and a QR Code to access the survey directly. To be eligible, all participants were ≥ 18 years old and resided in Brazil, and the questionnaire was completed to the last question. After the first weeks of boosting ads on social media, we noticed that the sample was primarily composed of women and people from South Brazil. To maintain the original sample target of a more representative sample concerning Brazilian regions and gender distribution, we changed the boosting strategy to reach more men and people from the other regions of the country.

### Instruments

The survey was available at the REDCap^®^ (Research Electronic Data Capture, Vanderbilt University, Nashville, TN, USA) online platform. The first page of the survey contained the informed consent form, followed by a button to accept the terms and begin answering the questionnaire. The complete survey was composed of 56 questions divided into four sections (see details in Supplementary Questionnaires S1–S4).

The first section (12 questions, Supplementary Questionnaire S1) was a close-ended survey used for collecting sociodemographic data; the second section (nine questions, Supplementary Questionnaire S2) revolved around social distancing during the pandemic. As there is no validated questionnaire for social distancing during pandemics, questions regarding social distancing were discussed by researchers and sent to external specialists in the field for a first evaluation and then tested out with a small pilot study in a group with similar characteristics to the target sample. The feedback sent by the specialists and non-specialists were used by the authors to adapt and improve the questionnaire and were not analyzed in a quantitative manner. The third section was composed of the Brazilian validated version of the Alcohol, Smoking and Substance Involvement Screening Test (ASSIST, version 3.1) instrument (24) (13 questions, Supplementary Questionnaire S3) to measure the use risk of psychoactive substances (tobacco, alcohol, cannabis, cocaine, amphetamines or ecstasy, hypnotics or sedatives, inhalants, hallucinogens, opioids or other drugs). The last section was related to mental health (21 questions, Supplementary Questionnaire S4) using the Depression, Anxiety and Stress Scale (DASS-21) scale, also validated in Portuguese (25). At the end of the survey, a counseling message based on a score calculated from their answers on the ASSIST was presented, with their risk of dependence being classified as low, moderate, or high. For moderate and high risk, participants were recommended to seek help from a health care practitioner.

### Measures

Sociodemographic data were collected in categories—except for age, which was a continuous variable. Ethnic groups were defined as “White” (Caucasian origins), “Black/Mixed” (African origins and mixed with other ethnic groups), and “Others" (Asians, native or indigenous, rather not answer). Social distancing perception regarded the level of restriction in social distancing considering six categories, which were grouped for some statistical analysis: “low” (1-not doing it and 2-very flexible), “medium” (3-flexible and 4-moderate), and “high” (5-rigorous and 6-very rigorous).

The change in drug consumption (primary outcome) was verified through an adapted ASSIST question: “In your personal perception, was the amount of your consumption of each substance increased, decreased, or maintained compared to the period before the pandemic?”. The ASSIST microstructured questionnaire included questions regarding self-reported frequency of drug use, lifetime use, and use in the last three months (which, in our study, consisted of the period during the first pandemic wave) and consequences related to use, based on DSM-IV criteria for drug dependence. The resulting score (secondary outcome) gives an indication of problematic use or not. From 0 to 3: occasional use (for alcohol: 0–10); ≥4 indicates moderate risk for dependence (for alcohol: 11–26); ≥27: suggestive of dependence or high risk of dependence (26).

The DASS-21 contemplated three subscales (depression, anxiety, and stress) with a Likert format, varying from 0 (did not apply to me at all) to 3 (applied to me very much or most of the time). Cutoffs for the three subscales are presented as “Normal” (depression: 0–9; anxiety: 0–7; stress: 0–14); “Mild” (depression: 10–13; anxiety: 8–9; stress: 15–18); “Moderate” (depression: 14–20; anxiety: 10–14; stress: 19–25); Severe (depression: 21–27; anxiety: 15–19; stress: 26–33); “Extremely severe” (depression: >28; anxiety: >20; stress: >34.

### Statistical analysis

Descriptive statistics were carried out using Excel^®^, and all other inferential analyses were run with IBM^®^ SPSS Statistics software (v20) or SigmaStat^®^ 3.1. The analysis of the ASSIST and DASS-21 scores according to social distancing or drug use alteration was carried out through a one-way ANOVA or Kruskal–Wallis test followed by Tukey's or Dunn's test when appropriate. Pearson's correlation test was used to verify associations between quantitative variables. The normality test performed was the Kolmogorov–Smirnov test, followed by the Equal Variance test to determine if it should be run as a parametric or a non-parametric test. Qualitative variables were analyzed through the chi-square test followed by residual analysis, considering the social distancing profile, according to each alteration in drug use. A *z*-test for proportions was performed for the comparison between increased vs. decreased consumption in the total population. For the confidence interval (CI_95%_) of the prevalence ratio, Wilson's score interval was calculated. To identify how much the severity of social distancing impacted drug use, a generalized linear model (GLM) was run for non-Gaussian probability distributions through linear, simple and multiple regression analysis. For this analysis, we kept the original stratification (1–6) of social distancing, since it presented power enough to maintain it. The scores for drug consumption characterized the dependent variable, while the severity of social distancing, as well as covariates of the sample profile, were defined as the independent variables of the model. For the identification of risks related to social distancing, the reference category chosen was “very rigorous”. The covariates defined as controls in the models were listed based on the significant results observed in the bivariate analyses (comparisons with the rigor of isolation with sociodemographic variables and DASS scale).

The F values, *t*-values, *z*-values, as well as the *N* and degrees of freedom numbers, are presented together with the values. Differences and associations were considered statistically significant when *P* ≤ 0.05. For the potential false-negative analysis, a power test was performed according to the hypothesis test used, considering a β ≥0.8 as an adequate result, and displayed each time the *P* value was less than.05. The sample size was calculated using the three most prevalent drugs in the target population (alcohol, tobacco and cannabis), comparing the prepandemic vs. pandemic alcohol use prevalence (main outcome).

## Results

The survey reached a total of 95,184 Facebook^®^ users (link clicks: 1,613), along with individuals reached through email and WhatsApp^®^. A total of 3,348 (3.5%) participants started to complete the questionnaire, and of those, 2,435 (2.6%) fully completed the survey and were used for the analyses. The majority of the participants were women (67.3%), white (74.6%), mostly single, divorced or widowed (65.2%), with completed higher education (63.7%), currently formally employed (32.6%), followed by studying (29.1%) as major occupations and a mean age of 32 (±10.8) years old.

**Figure 1:**
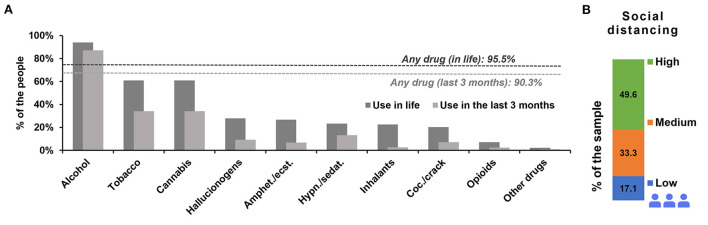
Prevalence of drug use in the lifetime and during the first pandemic wave. Dashed lines represent any drug use in the lifetime and any drug use during the first pandemic wave **(A)**. **(B)** Represents the percentage of social distancing perception from 2,435 participants of the survey among the sample during the pandemic period. The social distancing subsamples were as follows: low: *N* = 416; medium: *N* = 810; and high: *N* = 1,209. “Amphet.”: amphetamines; “ecst.”: ecstasy; “Coc.”: cocaine; “Hypn.”: hypnotics; “sedat.”: sedatives.

Overall lifetime drug use prevalence presented high percentages, showing that 95.5% (*N* = 2,325; 95% CI: 94.6–96.2%) of the participants had used any type of drug in their lifetime, and 90.3% (*N* = 2 198; 95% CI: 89.0–91.4%) had used any drug during the first pandemic wave or in the last three months (Figure 1A). Among the investigated drugs, alcohol was the most consumed either in the lifetime (94.0%; *N* = 2,288; 95% CI: 92.9–94.8%) or during the first pandemic wave (87.1%; *N* = 2,121; 95% CI: 85.7–88.4%). The most prevalent drugs were cannabis and tobacco, presenting lifetime use of 61.0% (*N* = 1,486; 95% CI: 59.1–63.0%) and 60.9% (*N* = 1,483; 95% CI: 58.9–62.8%), respectively, for which the use during the first pandemic wave was reported for tobacco as 34.2% (*N* = 833; 95% CI: 32.4–36.1%) and for cannabis as 34.1% (*N* = 830; 95% CI: 32.2–36.0%). Other substances represented 20–30% of use in the lifetime in the following order: hallucinogens (27.8%; *N* = 677; 95% CI: 26.1–29.6%), amphetamines/ecstasy (26.7%; *N* = 650; 95% CI: 25.0–28.5%), hypnotics/sedatives (23.3%; *N* = 568; 95% CI: 21.7–25.0%), inhalants (22.5%; *N* = 548; 95% CI: 20.9–24.2%), cocaine/crack (20.2%; *N* = 492; 95% CI: 18.7–21.8%), opioids (7.2%; *N* = 176; 95% CI: 6.3–8.3%), and others (2.2%; *N* = 53; 95% CI: 1.7–2.8%). Use during the first pandemic wave for these categories was much inferior than for alcohol, cannabis and tobacco; therefore, opioids, other drugs and injected drugs were grouped with those, so interpretation would be facilitated. Other substances presented lower percentages of use during the first pandemic wave in the following order: hallucinogens (9.3%; *N* = 227; 95% CI: 10.2–12.7%), amphetamines/ecstasy (6.7%; *N* = 162; 95% CI: 5.7–7.7%), hypnotics/sedatives (13.3%; *N* = 325; 95% CI: 12.1–14.8%), inhalants (2.5%; *N* = 61; 95% CI: 1.9–3.2%), cocaine/crack (7.2%; *N* = 175; 95% CI: 6.2–8.3%) and opioids (2.3%; *N* = 55; 95% CI: 1.7–2.9%), others (0.9%; *N* = 22; 95% CI:0.6–1.3%). All of the drugs presented relatively low use during the first pandemic wave compared to lifetime use, except alcohol and hypnotics/sedatives.

Concerning the social distancing profile for all participants, almost 50% (*N* = 1,209) perceived themselves to be in high social distancing, while 33.3% (*N* = 810) were in the medium social distancing level, and 17.1% (*N* = 416) were in the low social distancing level (Figure 1B). The drug with the highest percentage of drug use risk during the pandemic classified as high was alcohol (8.1%), and when adding high and medium risk together, the most prevalent class of drugs was hypnotics/sedatives (53%), followed by tobacco (52.7%), cannabis (49.3%), alcohol (42.4%), and the rest of the drugs presented less than 30% of medium plus high risk.

Table 1 displays the sociodemographic characteristics of the enrolled participants by substance group during the first pandemic wave. Although the data were organized from the most to least prevalent drug used during life, the prevalence of use during the first pandemic wave did not follow the same sequence, as observed in the discrimination percentages for each substance. Thus, the most consumed drugs during the first pandemic wave were alcohol, followed by hypnotics/sedatives, tobacco, cannabis, other drugs, cocaine/crack, hallucinogens, opioids, amphetamines/ecstasy, and inhalants. Considering the drug use in the last three months, differences in gender can be detected for several drugs. Men and “rather not answer” presented higher self-report for tobacco (men: *P* = 0.0124; “Rather not answer”: *P* = 0.0013), as well as women presented a lower frequency (women: *P* = 0.0019), with the same pattern for cannabis (men: *P* < 0.0001; “rather not answer”: *P* = 0.0010; women: *P* < 0.0001) and for hallucinogens (men: *P* = 0.0010; “rather not answer”: *P* = 0.0027; women: *P* < 0.0357). Meanwhile, for amphetamines/ecstasy, there was only a higher prevalence for “rather not answer” (*P* = 0.0010) and a lower prevalence for women (*P* = 0.0069), with the same being observed for inhalants (“rather not answer”: *P* = 0.0069; women: *P* = 0.0124). For ethnicity, “white” respondents showed lower use of inhalants (*P* = 0.0051) and cocaine/crack (*P* = 0.0014). Additionally, “other race” participants presented higher inhalant prevalence (*P* = 0.0215), and people who self-reported as the “black/mixed” ethnic group showed a higher prevalence of cocaine/crack use (*P* = 0.0014). Marital status showed differences for most drugs, excluding hypnotics/sedatives, opioids and others. Married individuals reported a lower perception of drug use (alcohol: *P* = 0.0069; tobacco: *P* < 0.0001; cannabis: *P* = 0.003; Hallucinogens: *P* = 0.0019; amphetamines/ecstasy: *P* = 0.0002; inhalants: *P* < 0.0001; cocaine/crack: *P* < 0.0001), while single/divorced/widower presented the opposite pattern (alcohol: *P* = 0.0124; tobacco: *P* < 0.0001; cannabis: *P* = 0.0003; hallucinogens: *P* = 0.0037; amphetamines/ecstasy: *P* = 0.003; inhalants: *P* < 0.0001; cocaine/crack: *P* < 0.0001). Education presented differences mostly for “higher education” and “incomplete higher education”, except for hypnotics/sedatives, opioids, and other drugs. Higher education presented lower use perception of tobacco (*P* < 0.0001), cannabis (*P* < 0.0001), hallucinogens (*P* < 0.0001), amphetamines/ecstasy (*P* < 0.0001), inhalants (*P* < 0.0001), and cocaine/crack (*P* < 0.0001), while incomplete higher education showed higher perception of use of the same drugs (tobacco, *P* < 0.0001; cannabis, *P* < 0.0001; hallucinogens, *P* < 0.0001; amphetamines/ecstasy, *P* < 0.0001; inhalants, *P* < 0.0001; cocaine/crack, *P* = 0.0010). Secondary education showed lower perception of alcohol use (*P* = 0.0002), as well as higher tobacco (*P* = 0.0037) use perception. Incomplete basic education demonstrated greater use of cannabis (*P* = 0.0124), hallucinogens (*P* = 0.0357), and cocaine/crack (*P* = 0.0215). Occupation before the pandemic exhibited a significant difference for informal job/self-employed individuals, who reported higher use of cannabis (*P* = 0.0001). Current occupation demonstrated a similar pattern, with a higher perception of cannabis use by the unemployed (*P* = 0.0069) and informal job/self-employed (*P* = 0.0455) individuals, while students (*P* = 0.0357) perceived a lower use of the same drug. It is important to note that alcohol users showed no difference for any of the categories of this variable. Household income before the pandemic showed clusters for groups with lower income and with higher income. Lower income groups, ranging from very low income to middle class, showed higher use perception of tobacco (income up to 750 R$: *P* = 0.0278; 751 up to 1,500 R$: *P* = 0.0037; 1,501 up to 3,000: *P* = 0.0019), cannabis (income up to 750 R$: *P* = 0.0069; 1,501 up to 3,000 R$: *P* < 0.0001) and hallucinogens (income up to 750 R$: *P* = 0.0215; 751 up to 1,500 R$: *P* = 0.0093). In higher-income groups, ranging from upper middle class to high class, cannabis use perception was lower (6,001 up to 9,000 R$: *P* = 0.0007), while for even higher income, tobacco (> 9,000 R$: *P* < 0.0001) and cannabis (> 9,000 R$: *P* < 0.0001), use perception was lower. Current household income showed similar results as before the pandemic, with lower income presenting a higher prevalence for tobacco (income up to 750 R$: *P* < 0.0001; 1,501 up to 3,000 R$: *P* = 0.0093) and cannabis (751 up to 1,500 R$: *P* = 0.0124; 1,501 up to 3.000 R$: *P* < 0.0001). On the other hand, higher income showed similar results to before the pandemic, showing lower use for tobacco (> 9,000 R$: *P* < 0.0001) and cannabis (6,001 up to 9,000 R$: *P* = 0.0019; > 9,000 R$: *P* < 0.0001). Income conditions before and during the pandemic indicated an overall higher use in lower income categories and lower use for higher income categories. Last, the mean ages for all groups ranged from 30.4 to 34.4 (SD varying from 12.4 to 17.4), and no significant difference was found when comparing these group means.

**Table 1:** Sociodemographic characteristics of participants according to substance use during the first pandemic wave.

**Variable**	**Categories**	**Total**	**Alcohol**	**Tobacco**	**Cannabis**	**Hallucin**.	**Amphet./ecst**.	**Hypn./sedat**.	**Inhalants**	**Coc./crack**	**Opioids**	**Others**
	Use in life: total N	2,435	2,288	1,483	1,486	677	650	568	548	492	176	53
	Use in the last	∑ -	2,121 (81.1)	833 (34.2)	830 (34.1)	227 (9.3)	162 (6.7)	325 (13.3)	61 (2.5)	175 (7.2)	55 (2.3)	22 (0.9)
	3 months	/ -	2,121 (92.7)	833 (56.2)	830 (55.9)	227 (33.5)	162 (24.9)	325 (57.2)	61 (11.1)	175 (35.6)	55 (31.3)	22 (41.5)
Gender	Female Male	1,637 (67.3) 783 (32.1)	1,421 (92.7) 687 (92.7)	501 (53.1)* 319 (60.5)*	456 (49.5)* 360 (65.5)*	99 (27.6)* 122 (39.5)*	74 (20.7)* 81 (28.6)	228 (60.0) 93 (50.8)	23 (8.0)* 35 (13.8)	79 (32.1) 91 (38.4)	30 (31.9) 23 (29.1)	10 (35.7) 10 (43.5)
Ehtnicity	White Black/mixed (black/white) Others	1,817 (74.6) 536 (22.0) 82 (3.4)	1,590 (92.7) 459 (92.9) 72 (92.3)	607 (54.9) 202 (62.3)* 24 (45.3)	600 (54.3) 192 (59.4) 38 (64.4)	163 (32.3) 54 (35.5) 10 (47.6)	114 (23.0) 39 (29.8) 9 (39.1)	244 (56.7) 68 (58.1) 13 (61.9)	35 (8.8)* 20 (15.6) 6 (26.1)*	113 (31.4)* 54 (48.2)* 8 (40.0)	40 (28.8) 12 (40.0) 3 (42.9)	15 (37.5) 5 (55.6) 2 (50.0)
Marital status	Married/stable union Single/divorced/widowed	838 (34.4) 1,587 (65.2)	711 (90.7)* 1,400 (93.7)*	202 (44.6)* 626 (61.2)*	204 (48.5)* 622 (58.8)*	44 (24.3)* 181 (36.8)*	22 (13.9)* 139 (28.5)*	92 (53.5) 233 (59.0)	7 (3.7)* 54 (15.0)*	33 (22.4)* 141 (41.2)*	16 (26.7) 39 (33.6)	6 (40.0) 16 (42.1)
Schooling	Higher education (HE) Incomplete HE Secondary education (SE) Incomplete SE	1,551 (63.7) 655 (26.9) 190 (7.8) 38 (1.6)	1,347 (93.1) 591 (93.7) 151 (85.8)* 31 (93.9)	385 (45.6)* 335 (71)* 94 (67.6)* 19 (67.9)	395 (47)* 336 (67.7)* 81 (63.3) 18 (81.8)*	86 (25.4)* 119 (44.2)* 15 (25.4) 7 (63.6)*	55 (16.9)* 91 (35.7)* 13 (21.7) 3 (33.3)	174 (57) 117 (57.6) 29 (55.8) 5 (62.5)	19 (6.3)* 37 (19.7)* 5 (9.8) 0 (0)	66 (26.8)* 80 (44.9)* 22 (37.9) 7 (70.0)*	26 (29.9) 25 (34.7) 4 (23.5) 0 (0)	12 (40.0) 8 (53.3) 2 (25.0) 0 (0)
Current occupation	Informal job/self employed Formal Employed Unemployed Student Retired Others	391 (16.1) 796 (32.6) 215 (8.8) 708 (29.1) 58 (2.4) 263 (10.8)	345 (92.2) 710 (94.9) 191 (91.8) 607 (91.7) 48 (90.6) 217 (90.4)	159 (61.2) 254 (54.4) 98 (61.3) 223 (53.9) 12 (44.4) 85 (55.6)	160 (61.5)* 247 (53.7) 111 (65.7)* 229 (51.7)* 4 (40.0) 77 (54.2)	49 (32.9) 63 (29.4) 28 (37.3) 69 (37.9) 0 (0) 17 (33.3)	37 (27.8) 41 (20.8) 22 (29.3) 52 (28.4) 0 (0) 9 (15.3)	49 (51) 106 (63.1) 36 (54.5) 91 (54.8) 9 (90.0) 32 (53.3)	13 (11.2) 20 (10.8) 9 (15.0) 14 (11.0) 0 (0) 4 (7.5)	32 (31.4) 61 (35.5) 35 (49.3) 33 (33.7) 1 (20.0) 12 (27.9)	12 (35.3) 15 (29.4) 9 (40.9) 11 (23.9) 0 (0) 7 (33.3)	4 (44.4) 3 (25.0) 4 (57.1) 5 (29.4) 0 (0) 5 (83.3)
Household income before pandemic	Without income Income up to 750 R$ 751 up to 1,500 R$ 1,501 up to 3,000 R$ 3,001 up to 6,000 R$ 6,001 up to 9,000 R$ > 9,000 No answer	29 (1.2) 40 (1.6) 198 (8.1) 518 (21.3) 638 (26.2) 341 (14.0) 584 (24.0) 27 (1.1)	26 (92.9) 36 (97.3) 171 (91.0) 459 (93.5) 564 (93.4) 293 (91.6) 502 (92.6) 22 (91.7)	10 (45.5) 22 (75.9)* 95 (67.9)* 225 (63.2)* 215 (56.9) 102 (52.6) 138 (44.4)* 10 (52.6)	13 (56.5) 24 (80.0)* 88 (62.0) 244 (67.4)* 217 (55.9) 87 (44.6)* 134 (43.8)* 10 (66.7)	6 (50.0) 11 (57.9)* 35 (46.7)* 63 (37.3) 50 (28.2) 20 (25.3) 37 (29.4) 3 (42.9)	1 (16.7) 8 (53.3) 21 (35.6) 43 (25.9) 33 (19.3) 23 (27.1) 28 (21.5) 2 (40.0)	3 (42.9) 5 (55.6) 34 (60.7) 77 (57.9) 93 (57.4) 32 (46.4) 69 (60.5) 5 (83.3)	0 (0) 2 (16.7) 8 (17.0) 11 (8.1) 16 (11.4) 7 (9.2) 14 (11.9) 1 (20.0)	3 (50.0) 6 (54.5) 21 (38.9) 54 (40.3) 47 (35.1) 17 (31.5) 22 (25.9) 2 (50.0)	0 (0) 0 (0) 8 (50.0) 10 (28.6) 15 (31.3) 9 (34.6) 10 (25.0) 2 (100)	0 (0) 1 (50.0) 3 (42.9) 5 (45.5) 6 (42.9) 1 (14.3) 4 (50.0) 0 (0)
Current household income	Without income Income up to 750 R$ 751 up to 1,500 R$ 1,501 up to 3,000 R$ 3,001 up to 6,000 R$ 6,001 up to 9,000 R$	36 (1.5) 52 (2.1) 253 (10.4) 551 (22.6) 588 (24.2) 334 (13.7)	33 (94.3) 47 (92.2) 216 (91.5) 498 (95.0) 510 (92.2) 288 (90.9)	17 (60.7) 28 (77.8)* 112 (61.9) 246 (64.9)* 186 (55.5) 95 (50.0)	20 (71.4) 28 (70.0) 117 (64.6)* 256 (66)* 185 (54.1) 84 (45.2)*	8 (53.3) 12 (48.0) 35 (41.2) 57 (31.5) 59 (36.2) 17 (22.7)	5 (45.5) 8 (44.4) 28 (34.6) 37 (22.3) 36 (22.8) 21 (25.0)	1 (12.5) 11 (61.1) 45 (65.2) 87 (55.4) 76 (58.0) 40 (54.8)	0 (0) 4 (22.2) 3 (6.4) 14 (9.6) 19 (13.8) 7 (10.4)	5 (38.5) 8 (47.1) 30 (44.8) 50 (37.6) 43 (35.5) 16 (29.6)	0 (0) 1 (16.7) 14 (56.0) 10 (25.6) 13 (30.2) 7 (30.4)	0 (0) 0 (0) 4 (57.1) 4 (33.3) 7 (53.8) 3 (33.3)
	> 9,000 R$ No answer	537 (22.0) 60 (2.5)	463 (93.2) 20 (95.2)	122 (43.3)* 11 (64.7)	116 (41.4)* 9 (60.0)	35 (31.0) 1 (16.7)	23 (20.0) 1 (25.0)	52 (55.9) 4 (80.0)	11 (10.5) 1 (16.7)	18 (25.4) 2 (50.0)	8 (23.5) 1 (100)	2 (40.0) 0 (0)
Age	Mean (±SD)	32.0 (10.8)	32.6 (12.4)	32.9 (12.4)	33.2 (12.9)	34.4 (14.6)	33.7 (13.5)	32.0 (13.2)	32.6 (13.6)	32.4 (13.8)	30.4 (12.9)	32.0 (17.4)

Presented as *N* (%). Percentages were calculated based on the total of each category, according to drug use during the first pandemic wave. The total number of respondents for each drug use corresponds to a different *N* according to the prevalence of use within those who have used the drug during their lifetime. Sociodemographic categorical variables were analyzed by the chi-square test followed by residual analysis. Blue bold values indicate a higher frequency, and red bold values indicate a lower frequency compared to the expected values (*P < 0.05). The average age between drug use groups was compared by one-way ANOVA (no difference found). Hallucin: hallucinogens; Amphet: amphetamines; Ecst: ecstasy; Hypn: hypnotics; Sedat: sedatives; Coc: cocaine. ∑Represents that the percentage was calculated using the total sample as the denominator (*N* = 2,435). / Indicates that the percentage was calculated using the *N* of each drug subgroup in the lifetime as the denominator.

### Hypothesis 1: Social distancing is associated with drug use changes during the COVID-19 pandemic

**Figure 2:**
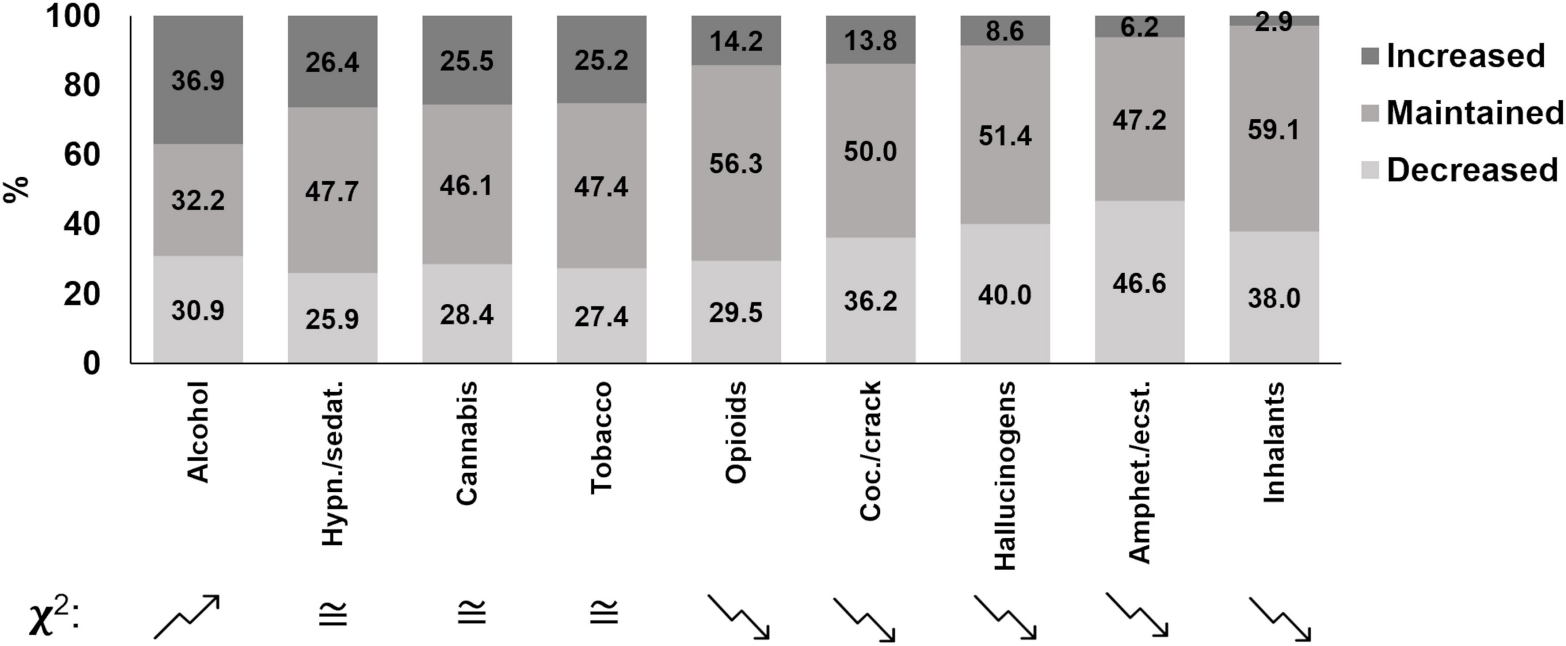
Percentage of self-reported changes in drug use during the first COVID-19 pandemic wave from survey participants in Brazil. Columns are ordered in a decreasing manner for increased proportions. A chi-square test for proportions was performed, comparing increased versus decreased proportions. Folded arrows indicate a significant difference between increased and decreased proportions; an upward folded arrow means it increased more than decreased, and a downward folded arrow means it decreased more than increased (chi square test; *P* < 0.001). ≅ means that there was no difference in the comparison between the decreased drug use group and the increased one. “Amphet.”: amphetamines; “ecst.”: ecstasy; “Coc.”: cocaine; “Hypn.”: hypnotics; “sedat.”: sedatives.

First, reported changes in drug consumption during the first pandemic wave, not considering the influence of social distancing, are presented through the percentage of drug users for each drug (Figure 2). This approach aims to display the general change in use of all drug users in this sample, making the social distancing effect on this sample clearer. Alcohol was the only substance that demonstrated an increase in use during the pandemic (*z* = 14.31, *P* < 0.0001), while a decrease was observed for opioids (*z* = 3.35, *P* = 0.001), cocaine/crack (*z* = 8.03, *P* < 0.0001), hallucinogens (*z* = 13.43, *P* < 0.0001), amphetamines/ecstasy (*z* = 16.49, *P* < 0.0001), and inhalants (*z* = 14.31, *P* < 0.0001). The drugs for which no general change was observed were hypnotics/sedatives (*z* = 0.14, *P* = 0.893, β = 0.03), cannabis (*z* = 1.74, *P* = 0.082, β = 0.41) and tobacco (*z* = 1.29 *P* = 0.196, β = 0.25).

**Figure 3:**
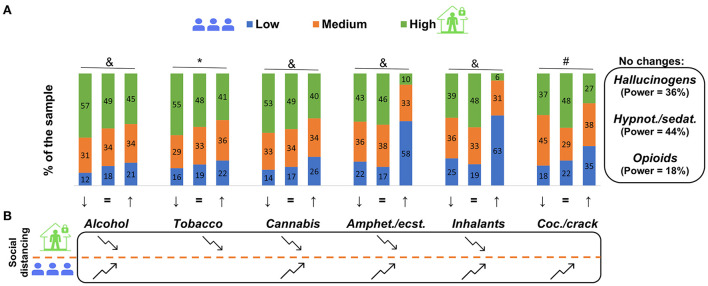
Change in drug use, according to social distancing perception levels, comparing before and during the COVID-19 pandemic from 2,435 survey participants in Brazil. In **(A)**, ↓, = and ↑ represent a decrease, no change and an increase in drug use, respectively. Piled columns represent the percentage of high, medium and low social distancing subgroups. *High social distancing individuals perceive greater reduction of use. ^#^Low social distancing individuals perceive a greater increase in use. ^&^High social distancing individuals perceive a greater increase in use, and low social distancing individuals perceive a greater reduction in use. Chi-square test, followed by residual analysis, *P* < 0.05. No changes box represents drugs that had no change when compared before and during the pandemic with the respective power test result **(A)**. **(B)** Represents a summary of the alterations found concerning the drug use changes of each drug. Folded arrows represent a decrease or increase in use according to the distancing perception level. Folded arrows pointing downward indicate that, for high social distancing, the prevalence of the reduction was higher and/or the prevalence of the increase was lower. Folded arrows pointing upward indicate that, for low social distancing, the prevalence of the increase was higher and/or the prevalence of the reduction was lower **(B)**. “Amphet.”: amphetamines; “ecst.”: ecstasy; “Coc.”: cocaine; “Hypn.”: hypnotics; “sedat.”: sedatives.

However, when analyzing the drug use changes according to social distancing (Figure 3), some evidence of its effect was found for alcohol ($\chi_{\left(4,\text{N}=2,288\right)}^{2}$ = 31.50; *P* < 0.001); tobacco ($\chi_{\left(4,\text{N}=1,483\right)}^{2}$ = 14.83; *P* = 0.005); cannabis ($\chi_{\left(4,\text{N}=1,486\right)}^{2}$ = 23.77; *P* < 0.001); amphetamines/ecstasy ($\chi_{\left(4,\text{N}=650\right)}^{2}$ = 38.06; *P* < 0.001); inhalants ($\chi_{\left(4,\text{N}=548\right)}^{2}$ = 21.11; *P* < 0.001); and cocaine/crack ($\chi_{\left(4,\text{N}=492\right)}^{2}$ = 21.03; *P* < 0.001). High social distancing participants reported a reduction in alcohol (*P* = 0.001), tobacco (*P* = 0.044), cannabis (*P* = 0.024), amphetamines/ecstasy (*P* = 0.002), and inhalants (*P* = 0.024) use, while low social distancing participants reported an increase in alcohol (*P* = 0.001), cannabis (*P* = 0.001), amphetamines/ecstasy (*P* < 0.0001), and cocaine/crack (*P* = 0.027) use. Three drug groups did not present statistically significant differences between the observed and expected frequencies, although with low statistical power: hallucinogens ($\chi_{\left(4,\text{N}=677\right)}^{2}$ = 4.69; *P* = 0.321; β = 0.36); hypnotics/sedatives ($\chi_{\left(4,\text{N}=568\right)}^{2}$ = 5.78; *P* = 0.216; β = 0.44); and opioids ($\chi_{\left(4,\text{N}=53\right)}^{2}$ = 0.56; *P* = 0.966; β = 0.18).

**Figure 4:**
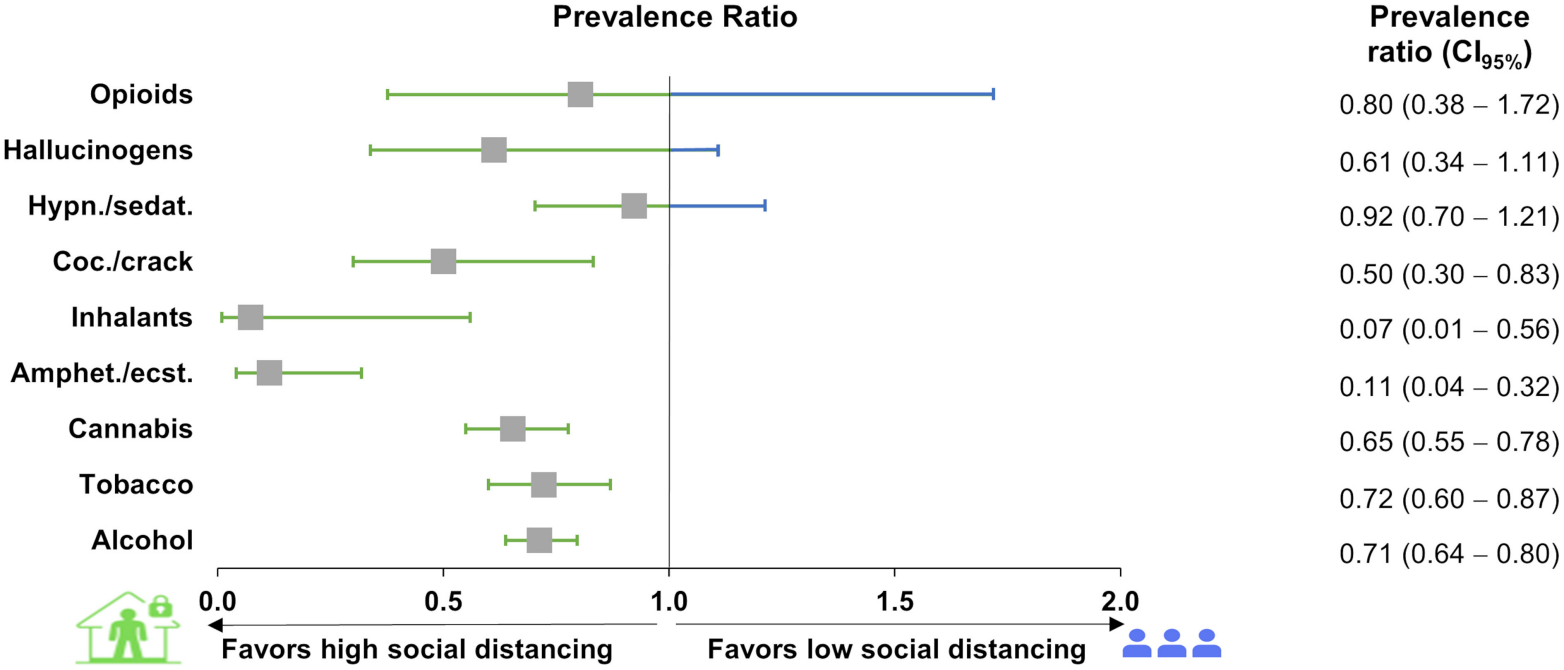
Prevalence ratio of changes in drug use and social distancing perception from 2,435 participants of the survey in Brazil during the COVID-19 pandemic. The prevalence ratio was calculated using the high (exposed group) and low (control group) social distancing individuals, excluding the medium social distancing individuals. Boxes represent the prevalence ratios, and lines represent the 95% CI. Prevalence ratios with 95% CIs are displayed. “Hypn.”: hypnotics; “sedat.”: sedatives; “Coc.”: cocaine; “Amphet.”: amphetamines; “ecst.”: ecstasy.

Another statistical strategy to analyze changes in drug use was to calculate the prevalence ratio considering low social distancing as the control compared to high social distancing and using the outcome as an increase and decrease in substance use perception (Figure 4). Six prevalence ratios (alcohol, tobacco, cannabis, amphetamines/ecstasy, inhalants, and cocaine/crack) showed significantly lower ratios for an increase in drug use perception and high social distancing.

### Hypothesis 2: Self-reported drug consumption changes are associated with mental health issues as well as sociodemographic factors

Table 1 presents the absolute and relative prevalence of the drugs used stratified by several characteristics. In summary, the following factors presented a higher prevalence for at least one of the drugs: male; black/mixed; single/divorced/widower; Southeast region; incomplete education and secondary education; informal job/self-employed and unemployed before or during the pandemic; and without income and income up to $3,000 R before or during the pandemic. The following factors presented lower prevalence for at least one of the drugs: female; married/stable union; higher education; formally employed, student, retired, and income of 3,001 up to > 9,000 R$.

**Figure 5:**
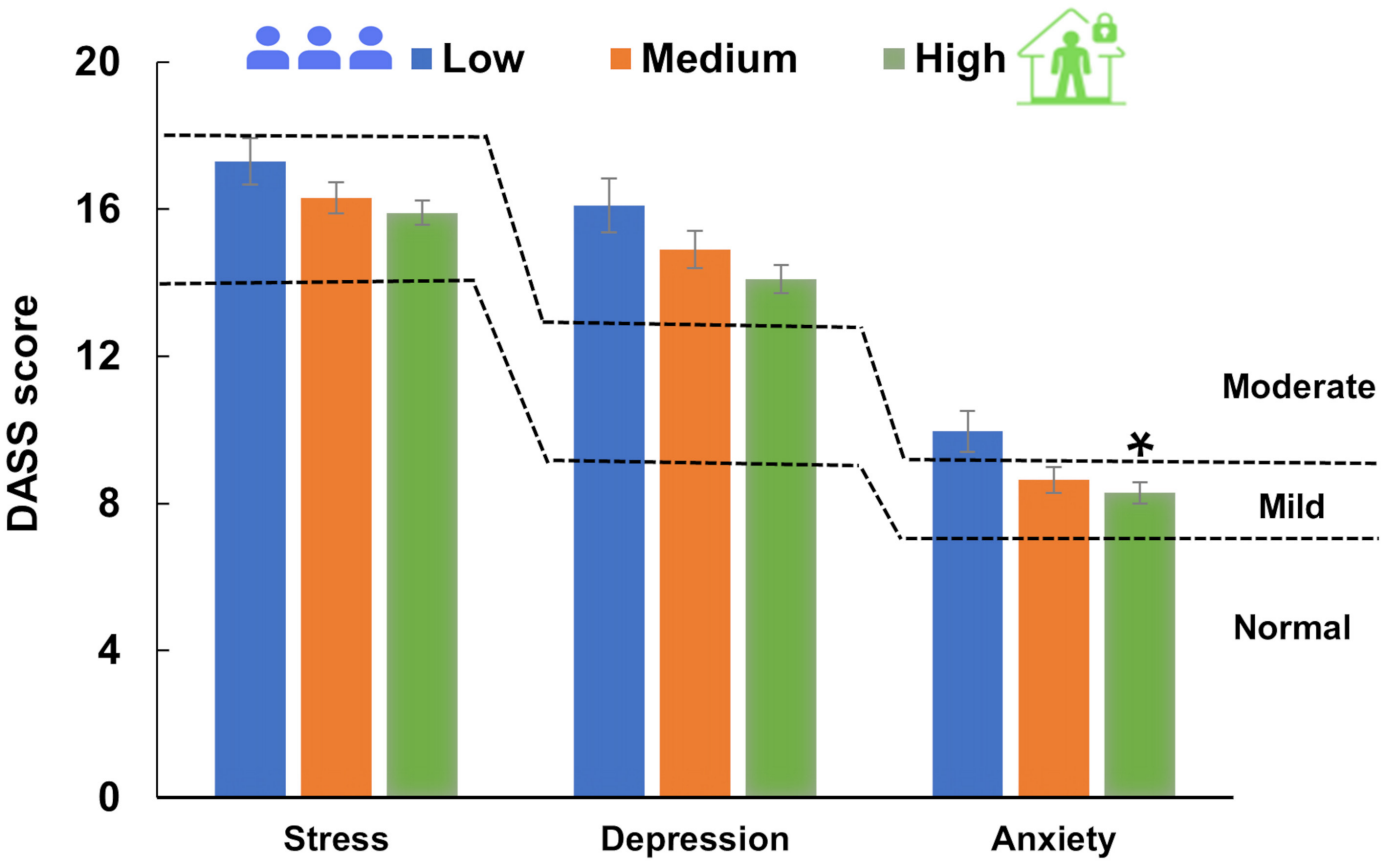
Social distancing and DASS-21 score (mean ± SEM). Dashed lines represent DASS-21 severity ratings of depression, anxiety and stress subscales. One-way ANOVA (depression) and one-way ANOVA on ranks (stress and anxiety) were performed. The means for each subscale were 16.3, 14.7 and 8.7 for stress, depression and anxiety, respectively. *Statistical difference in the ANOVA test (*P* = 0.017), showing a difference in the Tukey test between high and low social distancing (*P* = 0.012). Social distancing subsamples corresponded to low: *N* = 416; medium: *N* = 810; and high: *N* = 1,209.

The association between social distancing during the pandemic period and depression, anxiety, and stress self-reporting according to DASS-21 subscale scores is displayed in Figure 5. The means for each subscale for stress, depression and anxiety were 16.3, 14.7 and 8.7, respectively. A significant difference was found for anxiety scores (*F*_(2, 2, 146)_ = 4.07, *P* = 0.017), with high social distancing individuals presenting lower scores than low social distancing individuals (*q* = 4.03, *P* = 0.012), but no difference was found between medium social distancing vs. high social distancing: *q* = 1.08, *P* = 0.724; and low social distancing: *q* = 2.98, *P* = 0.089. Although the results are shown as the mean ± SEM in Figure 5, both depression and stress scores failed in the equal variance test (*P* < 0.05) and were carried out by a nonparametric test: depression (*H*_(2)_ = 4.91 *P* = 0.086, β = 0.51); and stress: (*H*_(2)_ = 3.47, *P* = 0.177, β = 0.18).

Several medium and weak positive correlations were observed between the ASSIST scores and the DASS scores, meaning that people with high stress, anxiety and depression scores also presented high scores on the ASSIST drug use scale (Table 2). Almost all variables presented associations with each other except for the number of individuals living in the same residence, which had only weak associations with all three DASS-21 scores (*r* < 0.1; *P* >0.05). The DASS-21, on the other hand, while demonstrating strong associations between depression, anxiety and stress scores, showed weak associations with all remaining variables. The higher *r*-values found were between opioids and hypnotics/sedatives (*r* = 0.712; *P* < 0.0001), opioids and hallucinogens (*r* = 0.550; *P* < 0.0001), opioids and inhalants (*r* = 0.441; *P* < 0.0001), and between hallucinogens and amphetamines/ecstasy (*r* = 0.613; *P* < 0.0001) and hallucinogens and inhalants (*r* = 0.466; *P* < 0.0001). Amphetamines/ecstasy also presented moderate associations with inhalants and cocaine/crack (*r* = 0.564 and 0.402, *P* < 0.0001, respectively). Other associations did not reach values higher than *r* = 0.4, although they were statistically significant.

**Table 2:**
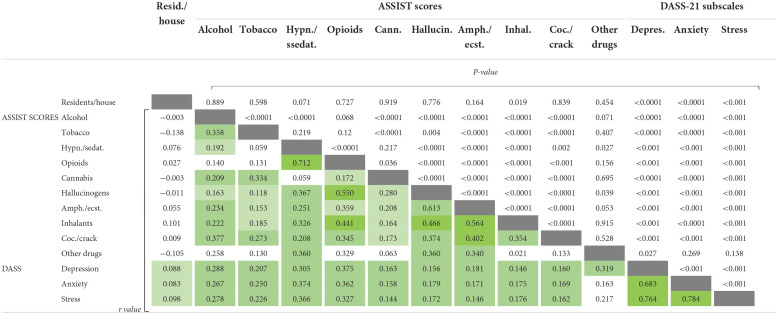
Associations between ASSIST scores, DASS-21 scores and residents per house variables.

Drug user groups were composed of the individuals who used any drug during their lifetime from all 2,435 participants of the survey in Brazil during the COVID-19 pandemic. ASSIST subscale scores represent drug use profiles during the first pandemic wave. At the bottom/left, Pearson correlation coefficients (r values) are presented in a green gradient. Color saturation (darker to lighter green) indicates correlation strength. At the top/right, P values are presented. “Cann.”: Cannabis; “Amph.”: amphetamines; “ecst.”: ecstasy; “Coc.”: cocaine; “Hypn.”: Hypnotics; “sedat.”: sedatives; “Depres.”: depression.

Finally, the generalized linear model helped in the expansion of knowledge on the impact of the severity of social distancing—considering the original six groups—on drug use and the relationship with sociodemographic and mental health data. A higher social distancing level greatly influenced drug use (alcohol, tobacco, cannabis and cocaine/crack) according to the degree of social distancing. Additionally, lower education status influenced the chance of using those drugs. The higher the household income, the higher the Tobacco ASSIST score; however, the higher the income, the lower the ASSIST scores for alcohol and cannabis. An additional influence of approximately 10% was seen for the depression score for all drugs considered herein. Male sex influenced the interaction between social distancing and drug use scores for alcohol and cannabis, and the younger age group presented a lower prevalence risk of alcohol use (Table 3). However, that statistical approach adds the depression state role to the drug use pattern. It must be emphasized that the results presented in Table 3 describe the drug use pattern during the first wave but do not explain the alteration of drug use compared to the period prior to the pandemic.

**Table 3:** Adjusted and unadjusted prevalence ratios reported for the drugs that presented statistical significance in the GLM test (alcohol; tobacco; cannabis; cocaine) in consumption, according to the ASSIST score during the COVID-19 pandemic, associated with social distancing perception level among a sample in Brazil.

**Variables**		**Prevalence ratio (CI**_**95%**_ **)**			
		**Alcohol** **(*N* = 2,274)**	**Tobacco** **(*N* = 1,483)**	**Cannabis** **(*N* = 1,486)**	**Coc./crack** **(*N* = 492)**
**Non-adjusted model**					
Social distancing level	1-Not doing it	8.00 (2.72; 23.22)^***^	-	43.56 (10.42; 172.03)^***^	136.44 (0.41; 1236.5)
	2-Very flexible	5.98 (1.08; 12.55)^***^	11.56 (0.76; 175.62)	41.19 (6.88; 108.17)^***^	32.27 (0.43; 40.32)
	3-Flexible	5.42 (2.57; 16.55)^***^	9.44 (1.07; 83.61)^*^	34.82 (18.29; 96.98)^***^	17.61 (1.47; 63.02)^*^
	4-Moderate	2.25 (1.02; 5.61)^***^	6.63 (1.06; 41.65)^*^	21.09 (4.00; 52.15)^***^	1.79 (0.06; 18.96)
	5-Rigorous	2.21 (1.16; 32.37)^***^	3.98 (0.65; 24.27)	6.70 (1.30; 34.60)^*^	0.36 (0.01; 10.71)
	6-Very rigorous	1	1	1	1
**Adjusted Model**					
Social distancing level	1-Not doing it	32.82 (6.54; 94.17)^***^	13.43 (5.93; 48.92)^**^	28.70 (11.32; 52.69)^**^	9.10 (0.03; 56.42)
	2-Very flexible	6.17 (8.44; 18.96)^***^	3.10 (0.22; 44.26)	11.94 (1.06; 134.15)^*^	15.46 (0.22; 64.89)
	3-Flexible	16.80 (7.15; 42.43)^***^	5.77 (0.70; 47.76)	17.51 (3.52; 42.11)^***^	12.42 (4.70; 30.88)^*^
	4-Moderate	18.09 (5.12; 63.90)^***^	3.41 (0.57; 20.24)	7.12 (1.47; 34.40)^*^	1.36 (0.05; 37.34)
	5-Rigorous	8.49 (2.47; 29.11)^***^	3.55 (0.62; 20.39)	4.69 (1.00; 21.93)^*^	0.32 (0.01; 8.71)
	6-Very rigorous	1	1	1	1
**Schooling**					
Incomplete SE		5.00 (2.66; 10.58)^**^	12.91 (0.33; 511.29)	19.42 (9.65; 37.25)^***^	11.42 (7.15; 27.49)^***^
Secondary Education (SE)		0.40 (0.10; 1.60)	7.75 (3.10; 18.66)^***^	12.95 (2.48; 21.68)^**^	3.78 (0.29; 48.71)
Incomplete HE		4.29 (1.88; 9.77)^***^	9.84 (5.80; 26.85)^***^	6.07 (2.66; 23.01)^***^	10.95 (1.99; 38.16)^**^
Higher education (HE)		1	1	1	1
**Gender**					
Male		1.71 (1.27; 4.44)^*^	—	3.86 (2.91; 7.54)^***^	—
Female		1	—	1	—
**Cur. household income scale**		0.42 (0.34; 0.53)^***^	0.47 (0.35; 0.65)^***^	0.36 (0.27; 0.48)^***^	—
**Depression DASS-21 Scale**		1.05 (1.02; 1.07)^***^	1.08 (1.04; 1.12)^***^	1.07 (1.00; 1.11)^*^	1.07 (1.01; 1.13)^*^
**Age (years)**		0.97 (0.94; 0.99)^*^	—	—	—

The other drugs did not present statistical significance in the GLM analysis and are not shown here. Cur, current; PR, prevalence ratio; CI, confidence interval; SE, secondary education; HE, higher education. Coc.—Cocaine Generalized linear model (GLM) adjusted for gender, marital status, age, schooling, current household income (scale from “0”-lowest to “7”-higher), DASS scales for anxiety, depression and stress.

*** Means *P* ≤ 0.001;

** *P* ≤ 0.01;

* *P* ≤ 0.05. “-”Means that the estimates are not shown due to a discrepancy of those numbers, on account of a lack of covariables adjustment related to social distancing level. “—”Means that some covariables did not sustain themselves in the GLM, meaning they lost relevance and were excluded from the model.

## Discussion

This survey's main target was to verify whether social distancing could affect changes in the Brazilian adult population's drug consumption. The main finding of the present work is that people who showed high social distancing self-reported their perception of decreased drug use, while people with low social distancing perceived an increase in drug use. However, those findings do not emerge if changes in drug use are analyzed without considering the social distancing pattern. Complementarily, ASSIST risk scores analysis presented a pattern of higher levels of social distancing associated with lower ASSIST scores, while lower social distancing showed higher ASSIST scores. This suggests that those two groups (low and high social distancing) presented similar drug use patterns before the pandemic, and during the pandemic, the low social distancing individuals increased, while the high social distancing individuals decreased consumption for most of the drug classes. The sample characteristics certainly have an impact on the outcome obtained and, even though the sample profile is not the same as the general population of Brazil, some factors are much more associated with drug use. Taking alcohol as an example, a previous national study pointed that 66.4% of a Brazilian sample used alcohol in their lifetime, which is different from what we observed in our study (27).

Another focal point of this survey was to identify factors associated with drug consumption behaviors. Despite higher DASS scores of anxiety, but not depression or stress, being initially associated with low social distancing levels, depression influenced the interaction between social distancing and drug-related problems. In addition, all ASSIST scores presented weak associations with anxiety, depression and stress DASS scores. Furthermore, some sociodemographic characteristics were associated with a higher prevalence of use before the pandemic (men, single/divorced/widowed, incomplete higher education, informal job/self-employed, and low income), with some of those risk factors no longer presenting this association during the pandemic. This suggests that anxiety may be a moderator of drug use changes and that the effect of the pandemic and social distancing was strong enough to equalize drug consumption according to certain factors, making the sociodemographic groups of drug users somehow similar in their drug use behaviors during stressful times such as in the setting of the pandemic (9, 28, 29).

Alcohol was the most prevalent drug used in the lifetime and during the pandemic, for which a rise in consumption perception was observed, mainly for those who reported lower levels of social distancing. In fact, other studies showed a decrease in the consumption associated with social restriction regulation in young people (30, 31), as well as in the general population (32). Even when anxiety and depression indicators were elevated, adults showed a reduction in heavy drinking, associated with lower socialization (33). During the most restrictive months of social isolation, a generalized alcohol intake reduction was observed in Italy (13) and the US (34). However, an extensive study in Europe showed that, from 21 countries evaluated, four decreased, two increased, and 15 presented no changes in the frequency of alcohol consumption during the pandemic (10). Many determinants could be pointed at for these different patterns of alteration in alcohol consumption, but surely one of the most critical determinants is social distancing. When all data were analyzed in a multifactorial basis, alcohol use risk kept presenting association with lower social distancing levels, lower education level, male gender, lower income, lower age and higher depression scores. In fact, slight differences were found for tobacco, cannabis and cocaine for these factors.

Tobacco was the only drug with a high prevalence of use that did not present differences in the ASSIST score according to social distancing levels, and even when not considering the social distancing factor, the percentages of people who decreased or increased tobacco use were almost the same. Nevertheless, it was observed that high social distancing individuals exhibited a slight decline in the use of tobacco products. Even nicotine-containing products presented decreased use in teenagers (35) and in the general population (36) during the pandemic. However, an extensive study in Europe showed that in nine countries, there was an increase in consumption, while none presented a reduction in tobacco product consumption (10). It is possible that people knew that smoking could lead to a worse prognosis (37), and thus smokers—even those who were not following social distancing recommendations—did not substantially increase their cigarette use (38). However, even when a reduction in tobacco consumption is detected, that change might not be persistent (13). All of these data suggest that, according to the population and pandemic time course when data were collected, the profile of consumption of nicotine products might differ between studies.

Cannabis was the third most used drug in general and the most prevalent among the illegal drugs in this study. There was no overall difference between the percentage of people who reduced or increased their use, as also shown by another self-report web-based survey in Europe (39). However, in the present population, a decrease in cannabis use for individuals with high social distancing and an increase in use among those reporting low social distancing were observed. Legal issues must be taken into account, since the differences seen here may be related to the fact that recreational marijuana use is illegal in Brazil. In the Netherlands, for comparison, there was an increased use of cannabis in response to COVID-19 lockdown regulations (40). One should also consider that different responses may occur depending on the period of the pandemic in which data were collected, since people tend to adapt their behavior as time passes. Additionally, changes in the perception of threat induced by the disease or protection brought by preventive or therapeutic measures that are currently in place may also play a role in their response.

Concerning the other drugs studied here, there were basically three patterns that emerged: (i) higher ASSIST scores for the individuals with lower social distancing (hallucinogens, amphetamine/ecstasy, hypnotics/sedatives, inhalants and cocaine/crack); (ii) a greater perception of decreased consumption during the pandemic in the general analysis (opioids, cocaine/crack, hallucinogens and amphetamine/ecstasy); and (iii) an increase in drug consumption for the higher social distancing participants and a decrease in drug consumption for the lower social distancing participants (amphetamine/ecstasy, inhalants and cocaine/crack). The exception was hypnotics/sedatives, for which no change was observed. One possible cause for this last observation is the fact that this is a group of substances that are mostly used as medicines, meaning that it requires retention of a prescription for their dispensing in Brazil. Nevertheless, the results of change in use perception during the pandemic vary according to the location studied. Many studies have reported a reduction in the use of a large number of different types of drugs, mainly illicit drugs (10, 14, 41). The main justification for that reduction in use was due to a decrease in social interactions (14), which could be translated in our study as high social distancing. Clearly, the use of hypnotic/sedative medications does not seem to be influenced by the pandemic when looking at the overall data, as social distancing was also not a factor that led to different patterns of changes in the use of these drugs. Therefore, there might have simply been no change in physician prescription of these agents during the pandemic, even though the ASSIST questionnaire indicates that it refers to the “nonclinical” use of the drugs evaluated. Additionally, although some people may be decreasing their use for a number of reasons, others may be using this resource to alleviate the stress/anxiety associated with the pandemic, as observed in adults (8) and in adolescents (35).

We did not find significant differences between high and low social distancing profiles when depression and stress scores were analyzed, although anxiety scores were significant. Nevertheless, there were some weak/medium associations between those three DASS-21 scores and drug use scores from the ASSIST questionnaire. Data obtained by Weerakoon et al. (42) indicate no association between changes in alcohol intake and depression symptoms in a sample very similar to the one obtained in this study. However, in another work, individuals who showed an increase in alcohol use also showed higher depression rates (43), and a coping mechanism for depression during the pandemic was associated with an increase in cannabis intake (21). Additionally, alcohol and tobacco intake was associated with depression, anxiety, and stress (22, 28).

An interesting result presented in this study is that participants who were single, divorced, or widowed exhibited higher intake of all drugs compared to married/stable union during the pandemic, with the exception of hypnotics/sedatives and opioids. The lifetime use analyses showed that such differences did not appear for alcohol and inhalants. That has also been shown by other studies, where not currently married individuals presented a higher risk of excessive alcohol consumption, associated with higher levels of anxiety and depression (15). In the same direction, living alone was associated with higher cigarette use (39).

A relevant determinant of drug use changes during the pandemic was financial and employment consequences caused by COVID-19 countermeasures. People reporting being unemployed/in informal jobs presented a higher prevalence than expected of alcohol intake in life; nonetheless, during the pandemic, this difference disappeared. Several studies identified that lower income and job loss issues aggravated drug intake and that higher income and job maintenance were associated with protective factors for the increase in alcohol, tobacco, cannabis, and opioid consumption (10, 39, 42, 44, 45). In contrast, higher income and socioeconomic status were associated with better knowledge of the disease, leading to higher anxiety and higher alcohol intake in women (46). Another aspect associated with income and drug use is that restrictive anti-COVID-19 measures impacted drug production differently depending on the substance its market values, as reviewed by di Trana et al. (47). This was also observed in Australia where there was a decrease in supply associated with an increase in drug prices (14).

Advanced levels of education demonstrate a protective influence on drug use, since individuals with incomplete higher education showed more drug consumption for several drugs—except alcohol—than individuals with complete higher education during the pandemic. Indeed, US individuals with complete or incomplete secondary education reported increased drug use prevalence to deal with pandemic-related stress (8). In addition, age seems to be critical for drug use change during the pandemic, most likely due to a higher age being related to more education, better jobs, and income conditions, which are well known for their protective influence on drug use (48), which in turn may be associated with financial stability and fewer job losses.

In addition, the multiple regression analysis reinforced some already described results and clarified the magnitude of the influence of schooling, gender, income, DASS-21 scores and age on drug use risks during the COVID-19 pandemic in the population analyzed in Brazil.

### Limitations

First, although widely accepted for observational studies on drug use epidemiology, online survey results may be impacted by memory bias, especially when evaluating the self-perception of drug use, and even more notably so when analyzing the profile of use during a distant moment in time. To mitigate that memory and observer bias, we did not ask about frequency or amounts of use in that particular question but only if there was any perceptual alteration in the consumption and ran a pilot survey with external specialists. Second, since we did not evaluate the interfactor analysis, so we could not verify some possible confounding factors, such as the influence of the alteration of one drug in another. An additional potential confounding issue that is implicit in cross-sectional design is causality, since social distancing and the drug use perception are collected at the same time and might be influencing each other. However, to attenuate that bias, GLM analysis was carried out. Fourth, being a self-selected sample, potential sample selection biases may lead to specific characteristics of respondents, such as excluding people who did not have access to the internet or heavy users of some drugs, thus limiting its generalizability. This also led to several analyses in our study in which we did not have large enough samples to allow for a statistical comparison but also overrepresentation of certain groups, such as women, whites, and people with a degree. Another implicit issue is that there were different social distancing rules applied in Brazilian states and cities, which could lead to less comparable results. To mitigate some of those confounders, we evaluated several sociodemographic characteristics of the sample and explored the alterations in the profile of use by applying different statistical analysis strategies. Finally, the pandemic periods are not exactly comparable among countries since they do not present the same regulations and commitment to social distancing. For these reasons, our results should be taken with caution, but further studies following the same sample are planned to minimize those biases and to verify the pattern of drug use in the postpandemic period.

## Conclusions

This study aimed to investigate the drug use profile and associated factors in the Brazilian population during the first wave of the COVID-19 pandemic. Our results indicate that individuals who had a low social distancing profile increased the use of several drugs, and individuals with a higher social distancing profile decreased their drug use. Moreover, anxiety symptoms were likely associated with low social distancing, which was associated with increased drug use, and depression was associated with a higher drug use pattern during the first pandemic wave. Additionally, sociodemographic factors were associated with higher drug use prevalence during the pandemic.

## Data availability statement

The raw data supporting the conclusions of this article will be made available by the authors, without undue reservation.

## Ethics statement

The studies involving human participants were reviewed and approved by National Ethics Committee and Ethics Committee of Research of the Federal University of Health Sciences of Porto Alegre (#4241378). The patients/participants provided their written informed consent to participate in this study.

## Author contributions

MN: conceptualization, data curation, formal analysis, investigation, methodology, project administration, supervision, validation, visualization, writing–original draft preparation, and writing–review and editing. NH: investigation, validation, visualization, writing–original draft preparation, and writing–review and editing. FA: conceptualization, data curation, formal analysis, investigation, methodology, and writing–review and editing. LI: writing–review and editing. HC: conceptualization, data curation, formal analysis, investigation, and writing–review and editing. LF: conceptualization, investigation, methodology, visualization, and writing–review and editing. RG: conceptualization, writing–original draft preparation, and writing–review and editing. HB: conceptualization, funding acquisition, investigation, methodology, project administration, supervision, and writing–review and editing. All authors contributed to the article and approved the submitted version.

## Funding

This research was funded by Conselho Nacional de Desenvolvimento Científico e Tecnológico (CNPq) and Coordenação de Aperfeiçoamento de Pessoal de Nível Superior (CAPES) (*n* 0613/2020/Processo 88881.506886/2020-01).

## Conflict of interest

The authors declare that the research was conducted in the absence of any commercial or financial relationships that could be construed as a potential conflict of interest.

## Publisher's note

All claims expressed in this article are solely those of the authors and do not necessarily represent those of their affiliated organizations, or those of the publisher, the editors and the reviewers. Any product that may be evaluated in this article, or claim that may be made by its manufacturer, is not guaranteed or endorsed by the publisher.
