# Supplementary material for: Social distancing and changes in drug use: Results from a cross-sectional study during the COVID-19 pandemic in Brazil
Source: Front Psychiatry. 2022 Nov 9;13:999372. doi: 10.3389/fpsyt.2022.999372 (PMC9682187; doi:10.3389/fpsyt.2022.999372)
Supplement: Supplementary file 1 [file Data_Sheet_1.pdf]

**Supplementary Material**

**Title:** Social distancing and changes in drug use: Results from a cross-sectional study during the COVID-19 pandemic in Brazil

**Running Head:** Drug Use Increase and Low Social Distancing

**Authors:** Maurício Schüler Nin PhD, Nubia Heidrich MSc, Felipe Borges Almeida MSc, Lucas dos Reis Izolan MSc, Hilda M. R. M. Constant PhD, Luana Freese PhD, Rosane Gomez PhD, Helena M. T. Barros

**Table of contents**

**Table S1**. STROBE Statement—Checklist of items that should be included in reports of cross-sectional studies. 2

**Questionnaire S1**. Informed consent and socio demographic data questionnaire 4

**Questionnaire S2**. Social distancing questionnaire. 8

**Questionnaire S3**. ASSIST questionnaire (validated in Portuguese) [45]. 9

**Questionnaire S4**. DASS-21 questionnaire (validated in Portuguese) [46]. 21

**Table S1.** STROBE Statement—Checklist of items that should be included in reports of cross-sectional studies.

| | Item No | Recommendation | Page No |
|---|---|---|---|
| **Title and abstract** | 1 | (*a*) Indicate the study's design with a commonly used term in the title or the abstract | M1 |
| | | (*b*) Provide in the abstract an informative and balanced summary of what was done and what was found | M2 |
| | | **Introduction** | |
| Background/ rationale | 2 | Explain the scientific background and rationale for the investigation being reported | M3 |
| Objectives | 3 | State specific objectives, including any prespecified hypotheses | M4 |
| | | **Methods** | |
| Study design | 4 | Present key elements of study design early in the paper | M4 |
| Setting | 5 | Describe the setting, locations, and relevant dates, including periods of recruitment, exposure, follow-up, and data collection | M4 |
| Participants | 6 | (*a*) Give the eligibility criteria, and the sources and methods of selection of participants | M4 |
| Variables | 7 | Clearly define all outcomes, exposures, predictors, potential confounders, and effect modifiers. Give diagnostic criteria, if applicable | M5 |
| Data sources/ measurement | 8 | For each variable of interest, give sources of data and details of methods of assessment (measurement). Describe comparability of assessment methods if there is more than one group | M5 |
| Bias | 9 | Describe any efforts to address potential sources of bias | M6 |
| Study size | 10 | Explain how the study size was arrived at | M7 |
| Quantitative variables | 11 | Explain how quantitative variables were handled in the analyses. If applicable, describe which groupings were chosen and why | M6 |
| Statistical methods | 12 | (*a*) Describe all statistical methods, including those used to control for confounding | M6 |
| | | (*b*) Describe any methods used to examine subgroups and interactions | M6, S14 |
| | | (*c*) Explain how missing data were addressed | & |
| | | (*d*) If applicable, describe analytical methods taking account of sampling strategy | - |
| | | (*e*) Describe any sensitivity analyses | - |
| | | **Results** | |
| Participants | 13 | (a) Report numbers of individuals at each stage of study—eg numbers potentially eligible, examined for eligibility, confirmed eligible, included in the study, completing follow-up, and analysed | M7 |
| | | (b) Give reasons for non-participation at each stage | - |

| | | | |
|---|---|---|---|
| | | (c) Consider use of a flow diagram | * |
| Descriptive data | 14 | (a) Give characteristics of study participants (eg demographic, clinical, social) and information on exposures and potential confounders | M8, S4 |
| | | (b) Indicate number of participants with missing data for each variable of interest | NA |
| Outcome data | 15 | Report numbers of outcome events or summary measures | M7-M16 |
| Main results | 16 | (*a*) Give unadjusted estimates and, if applicable, confounder-adjusted estimates and their precision (eg, 95% confidence interval). Make clear which confounders were adjusted for and why they were included | - |
| | | (*b*) Report category boundaries when continuous variables were categorized | - |
| | | (*c*) If relevant, consider translating estimates of relative risk into absolute risk for a meaningful time period | M16 |
| Other analyses | 17 | Report other analyses done—eg analyses of subgroups and interactions, and sensitivity analyses | S14 |
| **Discussion** | | | |
| Key results | 18 | Summarise key results with reference to study objectives | M17, M19-M20, S7-S12 |
| Limitations | 19 | Discuss limitations of the study, taking into account sources of potential bias or imprecision. Discuss both direction and magnitude of any potential bias | M20 |
| Interpretation | 20 | Give a cautious overall interpretation of results considering objectives, limitations, multiplicity of analyses, results from similar studies, and other relevant evidence | M17-M20, S13 |
| Generalisability | 21 | Discuss the generalisability (external validity) of the study results | M21 |
| **Other information** | | | |
| Funding | 22 | Give the source of funding and the role of the funders for the present study and, if applicable, for the original study on which the present article is based | M21 |

Note. M: manuscript's text page. NA: not applicable. *Considered not necessary. [&]No missing data since all questions were mandatory.

**Questionnaire S1**. Informed consent and socio demographic data questionnaire



# Avaliação continuada do consumo de álcool e outras drogas durante o isolamento social por COVID-19

Termo de Consentimento Livre e Esclarecido (TCLE)

Prezado participante,

Você está sendo convidado a participar do estudo "Avaliação continuada do consumo de álcool e outras drogas pelo isolamento social por COVID-19"! A previsão do tempo gasto total com esta pesquisa é de cerca de quinze minutos. Recomendamos que não inicie o preenchimento do questionário até que você tenha tempo suficiente para concluí-lo de uma só vez. Este é um estudo da Universidade Federal de Ciências da Saúde de Porto Alegre (UFCSPA) e da Universidade Federal do Rio Grande do Sul (UFRGS). Os dados serão coletados de forma anônima. Ao participar, você concorda que leu e entendeu todas as informações.

Sobre o que é este estudo e eu preciso participar?

A pandemia de COVID-19 está promovendo isolamento social, que pode estar associado ao aumento de risco de doenças psiquiátricas, além do aumento do risco de consumo de drogas de abuso. Neste sentido, esta pesquisa busca esclarecer se o fato de estar em isolamento social em decorrência da pandemia pode interferir no seu padrão de consumo de álcool, tabaco e/ou outras drogas. A participação é aberta a pessoas com 18 anos ou mais, que vivem no Brasil, é totalmente voluntária e não remunerada.

Quais são os benefícios e riscos de participar?

Benefícios: em curto prazo, você poderá receber um escore informativo sobre seu padrão de consumo atual após o preenchimento; em longo prazo, ajudar a resolver problemas decorrentes do isolamento social × consumo de substâncias a fim de estabelecer diretrizes de atendimento em caso de doença. Você será informado sobre formas de busca de ajuda por meio de contatos de grupos de autoajuda.

Riscos: você pode sentir-se desconfortável ao responder alguma pergunta, mas saiba que estas serão abordadas de forma anônima, mesmo que você complete seu e-mail para que possamos contatá-lo no próximo estudo. Se você sentir algum desconforto e desejar parar de responder, você é livre para fazê-lo, sem consequências.

Como meu questionário será armazenado e como será utilizado?

As respostas coletadas serão guardadas pelo grupo de pesquisa responsável, nas Universidades envolvidas, lembrando que são anônimas.

Assinando este termo você estará ciente que os dados coletados sejam utilizados para realização de pesquisa científica, sendo essas sempre condicionadas à aprovação ética dos órgãos competentes, mantendo a confidencialidade das informações e da identificação dos sujeitos. Os questionários e o banco de dados serão controlados por meio de sistemas de provedores classificados mediante senha de domínio somente dos pesquisadores envolvido no grupo de pesquisa.

Confirmo que recebi as informações:

1. Da garantia de receber resposta a qualquer pergunta ou esclarecimento a qualquer dúvida acerca dos riscos, benefícios e outros assuntos relacionados com a pesquisa;

2. Da liberdade de retirar meu consentimento, a qualquer momento, e deixar de participar do estudo;

3. Da garantia de que não serei identificado quando da divulgação dos resultados e que as informações utilizadas serão utilizadas apenas para fins científicos vinculados ao presente projeto de pesquisa;

4. De que os dados que estão sendo coletados armazenados por 5 anos no banco de dados Excel® e após este período serão excluídos permanentemente da rede.

5. De que o material colhido será acessado apenas pelos pesquisadores e os resultados, sem sua identificação, ser divulgado em eventos, revistas científicas e meios de comunicação.

5. Do compromisso de proporcionar informação atualizada obtida durante o estudo, ainda que esta possa afetar a minha vontade em continuar participando;

6. Pagamento: de que não terei nenhum tipo de despesa por participar deste estudo, bem como não receberei nenhum tipo de pagamento pela minha participação.

7. Ao clicar em aceito participar, concordarei com os termos acima e participando da pesquisa.

8. Ao clicar em não aceito, não poderei prosseguir para o questionário e não irei participar da pesquisa.

9. Estou ciente que, se houver algum dano decorrente da pesquisa, terei direito a solicitar indenização através das vias judiciais (Resolução CNS n°. 510/16, Artigo 17, Inciso IX).

Você pode contatar a pesquisadora responsável (Helena MT Barros) através do email helenbar@ufcspa.edu.br ou telefone (51) 3303-9000. Você também pode entrar em contato pelo email dedicado drogascovid19@gmail.com. Além disso, você pode contatar diretamente os comitês de ética em pesquisa (CEPs) das instituições.

CEP UFCSPA

Endereço: Rua Sarmento Leite, 245 (Prédio 3 / Sala 407), Porto Alegre / RS, CEP: 90050-170 / E-mail: cep@ufcspa.edu.br / Telefone: (51) 3303-8804

https://bvsms.saude.gov.br/bvs/saudelegis/cns/2013/res0466_12_12_2012.html

CEP UFRGS

Endereço: Av. Paulo Gama 110, Sala 311 / Prédio Anexo I da Reitoria - Porto Alegre / RS, CEP: 90040-060 / E-mail: etica@propesq.ufrgs.br / Telefone: (51) 3308-3738

Uma via termo será enviada a você por deste e-mail e a outra será arquivada pelo investigador principal.

Sua participação é muito importante para nós! Certifique-se que clica em "ENVIAR" ao final do preenchimento.

| | |
|---|---|
| Você aceita participar desse estudo? | ◯ Sim<br>◯ Não |
| Data de nascimento: | ______________________<br>(DD/MM/AAAA) |
| Qual seu sexo: | ◯ Feminino ◯ Masculino<br>◯ Prefiro não responder |
| A sua cor ou raça? | ◯ Branca ◯ Preta ◯ Amarela<br>◯ Parda ◯ Indígena ◯ Prefiro não responder |
| Qual é o seu estado civil? | ◯ Casado( a) ou união estável<br>◯ Divorciado(a), Desquitado (a) ou separado(a)<br>◯ Viúvo(a)<br>◯ Solteiro(a)<br>◯ Prefiro não responder |

| | |
|---|---|
| Qual estado você mora? | ◯ Acre (AC)<br>◯ Alagoas (AL)<br>◯ Amapá (AP)<br>◯ Amazonas (AM)<br>◯ Bahia (BA)<br>◯ Ceará (CE)<br>◯ Distrito Federal (DF)<br>◯ Espírito Santo (ES)<br>◯ Goiás (GO)<br>◯ Maranhão (MA)<br>◯ Mato Grosso (MT)<br>◯ Mato Grosso do Sul (MS)<br>◯ Minas Gerais (MG)<br>◯ Pará (PA)<br>◯ Paraíba (PB)<br>◯ Paraná (PR)<br>◯ Pernambuco (PE)<br>◯ Piauí (PI)<br>◯ Rio de Janeiro (RJ)<br>◯ Rio Grande do Norte (RN)<br>◯ Rio Grande do Sul (RS)<br>◯ Rondônia (RO)<br>◯ Roraima (RR)<br>◯ Santa Catarina (SC)<br>◯ São Paulo (SP)<br>◯ Sergipe (SE)<br>◯ Tocantins (TO) |
| Qual a cidade que você mora? | ______________________________ |
| Qual seu grau de instrução? | ◯ Analfabeto/Menos de um ano de instrução<br>◯ Fundamental Incompleto<br>◯ Fundamental Completo<br>◯ Ensino Médio Incompleto<br>◯ Ensino Médio Completo<br>◯ Superior Incompleto<br>◯ Superior Completo ou mais<br>◯ Prefiro não responder |
| Que opção melhor descreve sua situação de emprego atual? | ◯ Trabalhador informal/autônomo<br>◯ Trabalhador registrado/CLT<br>◯ Desempregado<br>◯ Dona-de-casa/do lar<br>◯ Estudante<br>◯ Aposentado<br>◯ Com incapacidade temporária ou em auxílio doença<br>◯ Com incapacidade permanente<br>◯ Outro<br>◯ Não quis responder |
| Qual é, aproximadamente, a sua renda (a soma da renda mensal de todos os que moram no seu domicílio)? | ◯ Sem renda<br>◯ Até R$ 750,00<br>◯ De R$ 751,00 até 1.500,00<br>◯ De R$ 1.501,00 até R$ 3.000,00<br>◯ De R$ 3.001,00 até R$ 6.000,00<br>◯ De R$ 6.001,00 até R$ 9.000,00<br>◯ Mais de R$9.000,00<br>◯ Não sei<br>◯ Prefiro não responder |

| | |
|---|---|
| Que opção melhor descreve sua situação de emprego antes da pandemia? | ◯ Trabalhador informal/autônomo<br>◯ Trabalhador registrado/CLT<br>◯ Desempregado<br>◯ Dona-de-casa/do lar<br>◯ Estudante<br>◯ Aposentado<br>◯ Com incapacidade temporária ou em auxílio doença<br>◯ Com incapacidade permanente<br>◯ Outro<br>◯ Não quis responder |
| Qual era, aproximadamente, a sua renda (a soma da renda mensal de todos os que moram no seu domicílio) antes da pandemia? | ◯ Sem renda<br>◯ Até R$ 750,00<br>◯ De R$ 751,00 até 1.500,00<br>◯ De R$ 1.501,00 até R$ 3.000,00<br>◯ De R$ 3.001,00 até R$ 6.000,00<br>◯ De R$ 6.001,00 até R$ 9.000,00<br>◯ Mais de R$9.000,00<br>◯ Não sei<br>◯ Prefiro não responder |

**Questionnaire S2**. Social distancing questionnaire.



| **Perguntas sobre Isolamento Social pela pandemia do novo coronavírus.** | |
|---|---|
| 1. Como você avalia o rigor/a flexibilidade com que você tem cumprido o isolamento social? | ◯ Não estou fazendo ◯ Muito flexível<br>◯ Flexível ◯ Médio ◯ Rigoroso<br>◯ Muito rigoroso |
| 2. Você já foi/está contaminado pelo "coronavírus"? | ◯ Não<br>◯ Não sei<br>◯ Sim, e estou plenamente recuperado<br>◯ Sim, e estou com sintomas leves<br>◯ Sim, e estou com sintomas preocupantes |
| 3. Como você avalia a intensidade de seu medo de se contaminar pelo "coronavírus"? | ◯ Não sinto medo<br>◯ Sinto pouco medo<br>◯ Sinto medo<br>◯ Sinto muito medo<br>◯ Tenho medo de morrer se me contaminar |
| 4. Durante a quarentena, você: | ◯ Permanece morando nas mesmas condições em que estava antes de se isolar<br>◯ Está morando em condição distinta daquela em que estava antes de se isolar |
| 5. Há quantas pessoas morando no domicílio em que você está passando a quarentena, contando com você? | ◯ 0 ◯ 1 ◯ 2 ◯ 3<br>◯ 4 ◯ 5 ◯ 6 ◯ 7<br>◯ 8 ◯ 9 ◯ 10 ◯ mais de 10 |
| 6. Você é profissional de saúde? | ◯ Sim<br>◯ Não |
| 7. Se sim, trabalha diretamente com a COVID-19? | ◯ Sim<br>◯ Não |
| 8. Qual sua categoria profissional? | ◯ Médico(a)<br>◯ Enfermeiro(a)<br>◯ Técnico(a) / auxiliar de enfermagem<br>◯ Fisioterapeuta(a)<br>◯ Farmacêutico(a)<br>◯ Psicólogo(a)<br>◯ Biomédico(a)<br>◯ outro |
| 9. Você acha que alterou o seu consumo de álcool durante a pandemia? | ◯ Não se alterou<br>◯ Sim, diminuiu<br>◯ Sim, aumentou |

A partir deste momento estaremos fazendo algumas perguntas sobre o seu consumo de Álcool, Tabaco e/ou outras drogas. Não é comum, mas se você perceber que está com vontade de usar alguma substância, sugerimos que você pare de responder o questionário, respire fundo e pense em algumas estratégias que podem lhe auxiliar. Se você quiser, pode escrever em um papel estas estratégias e visualizá-lo quando sentir necessidade. Exemplos destas estratégias são: telefonar para alguém que lhe dê apoio em não utilizar a(s) substância(s); pensar que o uso destas substâncias deixou você e as pessoas que você ama tristes. Se mesmo assim você não se sentir confortável, lembramos que você poderá interromper o preenchimento a qualquer momento.
Além disso, se for do seu interesse, após o término do questionário, iremos disponibilizar links de reunião online de grupo de autoajuda como Alcoólicos Anônimos e Narcóticos Anônimos.

**Questionnaire S3**. ASSIST questionnaire (validated in Portuguese) (24).



**Na sua vida (incluindo durante seu tempo de escola) qual(is) dessa(s) substâncias você já usou? (somente uso NÃO prescrito pelo médico)**

| | Não | Sim |
|---|---|---|
| Derivados do tabaco | ○ | ○ |
| Bebidas alcoólicas | ○ | ○ |
| Maconha | ○ | ○ |
| Cocaína, crack | ○ | ○ |
| Anfetaminas ou êxtase | ○ | ○ |
| Inalantes | ○ | ○ |
| Hipnóticos/sedativos | ○ | ○ |
| Alucinógenos | ○ | ○ |
| Opioides | ○ | ○ |
| Outras (descrever) | ○ | ○ |

Diga qual outra substância você utiliza: ______________________________

Para sua referência, estão listados abaixo os nomes comuns e comerciais das substâncias questionadas:

Tabaco: cigarro, charuto, cachimbo, fumo de corda, fumo, palheiro

Álcool: cerveja, vinho, champanhe, licor, pinga, uisque, vodca, vermutes, caninha, rum, tequila, gim

Maconha: baseado, erva, liamba, diamba, birra, fuminho, fumo, mato, bagulho, pango, manga-rosa, massa, haxixe, skank

Cocaína/crack: coca, pó, branquinha, nuvem, farinha, neve, pedra, cachimbo, brilho

Anfetaminas ou êxtase: bolinhas, rebites, bifetamina, moderine, MDMA, bala, Ritalina®

Inalantes: solventes, cola de sapateiro, tinta, esmalte, corretivo, verniz, tiner, clorofórmio, tolueno, gasolina, éter, lança-perfume, cheirinho da loló

Hipnóticos/sedativos: ansiolíticos, tranquilizantes, barbitúricos, fenobarbital, pentobarbital, benzodiazepínicos, diazepam, Rivotril®, Zolpidem®, Dormonid®

Alucinógenos: LSD, chá de lírio, ácido, passaporte, mescalina, peiote, cacto, Ayahuasca, Chá de Santo Daime, de cogumelo, "figurinha"

Opioides: morfina, codeína, ópio, heroína, elixir, metadona

Nas próximas questões, perguntaremos sobre o seu uso da(s) substância(s) assinaladas na pergunta anterior durante os últimos três meses (já durante a pandemia de COVID-19).

**Durante os três últimos meses, com que frequência você utilizou essa(s) substância(s) que mencionou?**

| | Nunca | 1 ou 2 vezes | Mensalmente | Semanalmente | Diariamente ou quase todos os dias |
|---|---|---|---|---|---|
| Derivados do tabaco | ○ | ○ | ○ | ○ | ○ |
| Bebidas alcoólicas | ○ | ○ | ○ | ○ | ○ |
| Maconha | ○ | ○ | ○ | ○ | ○ |
| Cocaína, crack | ○ | ○ | ○ | ○ | ○ |
| Anfetaminas ou êxtase | ○ | ○ | ○ | ○ | ○ |
| Inalantes | ○ | ○ | ○ | ○ | ○ |
| Hipnóticos/sedativos | ○ | ○ | ○ | ○ | ○ |
| Alucinógenos | ○ | ○ | ○ | ○ | ○ |
| Opioides | ○ | ○ | ○ | ○ | ○ |
| Outras | ○ | ○ | ○ | ○ | ○ |

**Durante os três últimos meses, com que frequência você teve um forte desejo ou urgência em consumir?**

| | Nunca | 1 ou 2 vezes | Mensalmente | Semanalmente | Diariamente ou quase todos os dias |
|---|---|---|---|---|---|
| Derivados do tabaco | ○ | ○ | ○ | ○ | ○ |
| Bebidas alcoólicas | ○ | ○ | ○ | ○ | ○ |
| Maconha | ○ | ○ | ○ | ○ | ○ |
| Cocaína, crack | ○ | ○ | ○ | ○ | ○ |
| Anfetaminas ou êxtase | ○ | ○ | ○ | ○ | ○ |
| Inalantes | ○ | ○ | ○ | ○ | ○ |
| Hipnóticos/sedativos | ○ | ○ | ○ | ○ | ○ |
| Alucinógenos | ○ | ○ | ○ | ○ | ○ |
| Opioides | ○ | ○ | ○ | ○ | ○ |
| Outras | ○ | ○ | ○ | ○ | ○ |

**Durante os três últimos meses, com que frequência o seu consumo desta(s) substância(s) resultou em problema de saúde, social, legal ou financeiro?**

| | Nunca | 1 ou 2 vezes | Mensalmente | Semanalmente | Diariamente ou quase todos os dias |
|---|---|---|---|---|---|
| Derivados do tabaco | ○ | ○ | ○ | ○ | ○ |
| Bebidas alcoólicas | ○ | ○ | ○ | ○ | ○ |
| Maconha | ○ | ○ | ○ | ○ | ○ |
| Cocaína, crack | ○ | ○ | ○ | ○ | ○ |
| Anfetaminas ou êxtase | ○ | ○ | ○ | ○ | ○ |
| Inalantes | ○ | ○ | ○ | ○ | ○ |
| Hipnóticos/sedativos | ○ | ○ | ○ | ○ | ○ |
| Alucinógenos | ○ | ○ | ○ | ○ | ○ |
| Opioides | ○ | ○ | ○ | ○ | ○ |
| Outras | ○ | ○ | ○ | ○ | ○ |

**Durante os três últimos meses, com que frequência, por causa do seu uso desta(s) substância(s), você deixou de fazer coisas que eram normalmente esperadas de você?**

| | Nunca | 1 ou 2 vezes | Mensalmente | Semanalmente | Diariamente ou quase todos os dias |
|---|---|---|---|---|---|
| Derivados do tabaco | ○ | ○ | ○ | ○ | ○ |
| Bebidas alcoólicas | ○ | ○ | ○ | ○ | ○ |
| Maconha | ○ | ○ | ○ | ○ | ○ |
| Cocaína, crack | ○ | ○ | ○ | ○ | ○ |
| Anfetaminas ou êxtase | ○ | ○ | ○ | ○ | ○ |
| Inalantes | ○ | ○ | ○ | ○ | ○ |
| Hipnóticos/sedativos | ○ | ○ | ○ | ○ | ○ |
| Alucinógenos | ○ | ○ | ○ | ○ | ○ |
| Opioides | ○ | ○ | ○ | ○ | ○ |
| Outras | ○ | ○ | ○ | ○ | ○ |

**Há amigos, parentes ou outra pessoa que tenha demonstrado preocupação com seu uso desta(s) substância(s)?**

| | NAO, nunca | SIM, nos últimos 3 meses | SIM, mas não nos últimos 3 meses |
|---|---|---|---|
| Derivados do tabaco | ○ | ○ | ○ |
| Bebidas alcoólicas | ○ | ○ | ○ |
| Maconha | ○ | ○ | ○ |
| Cocaína, crack | ○ | ○ | ○ |
| Anfetaminas ou êxtase | ○ | ○ | ○ |
| Inalantes | ○ | ○ | ○ |
| Hipnóticos/sedativos | ○ | ○ | ○ |
| Alucinógenos | ○ | ○ | ○ |
| Opioides | ○ | ○ | ○ |
| Outras | ○ | ○ | ○ |

**Alguma vez você já tentou controlar, diminuir ou parar o uso desta(s) substância(s) e não conseguiu?**

| | NÃO, nunca | SIM, nos últimos 3 meses | SIM, mas não nos últimos 3 meses |
|---|---|---|---|
| Derivados do tabaco | ○ | ○ | ○ |
| Bebidas alcoólicas | ○ | ○ | ○ |
| Maconha | ○ | ○ | ○ |
| Cocaína, crack | ○ | ○ | ○ |
| Anfetaminas ou êxtase | ○ | ○ | ○ |
| Inalantes | ○ | ○ | ○ |
| Hipnóticos/sedativos | ○ | ○ | ○ |
| Alucinógenos | ○ | ○ | ○ |
| Opioides | ○ | ○ | ○ |
| Outras | ○ | ○ | ○ |

Resultado escore tabaco

________________________________
(0-3: Nenhuma intervenção, 4-26: Intervenção breve, >27: Encaminhar para tratamento mais intensivo)

Resultado escore álcool

________________________________
(0-3: Nenhuma intervenção, 4-26: Intervenção breve, >27: Encaminhar para tratamento mais intensivo)

Resultado escore maconha

________________________________
(0-3: Nenhuma intervenção, 4-26: Intervenção breve, >27: Encaminhar para tratamento mais intensivo)

Resultado escore cocaína

________________________________
(0-3: Nenhuma intervenção, 4-26: Intervenção breve, >27: Encaminhar para tratamento mais intensivo)

Resultado escore anfetaminas

________________________________
(0-3: Nenhuma intervenção, 4-26: Intervenção breve, >27: Encaminhar para tratamento mais intensivo)

Resultado escore inalantes

________________________________
(0-3: Nenhuma intervenção, 4-26: Intervenção breve, >27: Encaminhar para tratamento mais intensivo)

| | |
|---|---|
| Resultado escore hipnóticos | __________________________________<br>(0-3: Nenhuma intervenção, 4-26: Intervenção breve, >27: Encaminhar para tratamento mais intensivo) |
| Resultado escore alucinógenos | __________________________________<br>(0-3: Nenhuma intervenção, 4-26: Intervenção breve, >27: Encaminhar para tratamento mais intensivo) |
| Resultado escore opioides | __________________________________<br>(0-3: Nenhuma intervenção, 4-26: Intervenção breve, >27: Encaminhar para tratamento mais intensivo) |
| Resultado escore outras | __________________________________<br>(0-3: Nenhuma intervenção, 4-26: Intervenção breve, >27: Encaminhar para tratamento mais intensivo) |
| Alguma vez você já usou drogas por injeção? (Apenas uso não médico) | ◯ NÃO, nunca<br>◯ SIM, nos últimos 3 meses<br>◯ SIM, mas não nos últimos 3 meses |

A partir de agora, perguntaremos sobre o seu uso desta(s) substância(s) durante os três meses ANTERIORES À PANDEMIA DE COVID-19 (dezembro de 2019 a fevereiro de 2020).

**No período anterior à pandemia de COVID-19, com que frequência você utilizou essa(s) substância(s) que mencionou anteriormente?**

| | Nunca | 1 ou 2 vezes | Mensalmente | Semanalmente | Diariamente ou quase todos os dias |
|---|---|---|---|---|---|
| Derivados do tabaco | ○ | ○ | ○ | ○ | ○ |
| Bebidas alcoólicas | ○ | ○ | ○ | ○ | ○ |
| Maconha | ○ | ○ | ○ | ○ | ○ |
| Cocaína, crack | ○ | ○ | ○ | ○ | ○ |
| Anfetaminas ou êxtase | ○ | ○ | ○ | ○ | ○ |
| Inalantes | ○ | ○ | ○ | ○ | ○ |
| Hipnóticos/sedativos | ○ | ○ | ○ | ○ | ○ |
| Alucinógenos | ○ | ○ | ○ | ○ | ○ |
| Opioides | ○ | ○ | ○ | ○ | ○ |
| Outras | ○ | ○ | ○ | ○ | ○ |

**No período anterior à pandemia de COVID-19, com que frequência você teve um forte desejo ou urgência em consumir?**

| | Nunca | 1 ou 2 vezes | Mensalmente | Semanalmente | Diariamente ou quase todos os dias |
|---|---|---|---|---|---|
| Derivados do tabaco | ○ | ○ | ○ | ○ | ○ |
| Bebidas alcoólicas | ○ | ○ | ○ | ○ | ○ |
| Maconha | ○ | ○ | ○ | ○ | ○ |
| Cocaína, crack | ○ | ○ | ○ | ○ | ○ |
| Anfetaminas ou êxtase | ○ | ○ | ○ | ○ | ○ |
| Inalantes | ○ | ○ | ○ | ○ | ○ |
| Hipnóticos/sedativos | ○ | ○ | ○ | ○ | ○ |
| Alucinógenos | ○ | ○ | ○ | ○ | ○ |
| Opioides | ○ | ○ | ○ | ○ | ○ |
| Outras | ○ | ○ | ○ | ○ | ○ |

**No período anterior à pandemia de COVID-19, com que frequência o seu consumo desta(s) substância(s) resultou em problema de saúde, social, legal ou financeiro?**

| | Nunca | 1 ou 2 vezes | Mensalmente | Semanalmente | Diariamente ou quase todos os dias |
|---|---|---|---|---|---|
| Derivados do tabaco | ○ | ○ | ○ | ○ | ○ |
| Bebidas alcoólicas | ○ | ○ | ○ | ○ | ○ |
| Maconha | ○ | ○ | ○ | ○ | ○ |
| Cocaína, crack | ○ | ○ | ○ | ○ | ○ |
| Anfetaminas ou êxtase | ○ | ○ | ○ | ○ | ○ |
| Inalantes | ○ | ○ | ○ | ○ | ○ |
| Hipnóticos/sedativos | ○ | ○ | ○ | ○ | ○ |
| Alucinógenos | ○ | ○ | ○ | ○ | ○ |
| Opioides | ○ | ○ | ○ | ○ | ○ |
| Outras | ○ | ○ | ○ | ○ | ○ |

**No período anterior à pandemia de COVID-19, com que frequência, por causa do seu uso desta(s) substância(s), você deixou de fazer coisas que eram normalmente esperadas de você?**

| | Nunca | 1 ou 2 vezes | Mensalmente | Semanalmente | Diariamente ou quase todos os dias |
|---|---|---|---|---|---|
| Derivados do tabaco | ○ | ○ | ○ | ○ | ○ |
| Bebidas alcoólicas | ○ | ○ | ○ | ○ | ○ |
| Maconha | ○ | ○ | ○ | ○ | ○ |
| Cocaína, crack | ○ | ○ | ○ | ○ | ○ |
| Anfetaminas ou êxtase | ○ | ○ | ○ | ○ | ○ |
| Inalantes | ○ | ○ | ○ | ○ | ○ |
| Hipnóticos/sedativos | ○ | ○ | ○ | ○ | ○ |
| Alucinógenos | ○ | ○ | ○ | ○ | ○ |
| Opioides | ○ | ○ | ○ | ○ | ○ |
| Outras | ○ | ○ | ○ | ○ | ○ |

**Na sua percepção pessoal, a quantidade de seu consumo dessa(s) substância(s) aumentou ou diminuiu entre o período anterior à pandemia e o período atual?**

| | Aumentou | Não mudou | Diminuiu |
|---|---|---|---|
| Derivados do tabaco | ○ | ○ | ○ |
| Bebidas alcoólicas | ○ | ○ | ○ |
| Maconha | ○ | ○ | ○ |
| Cocaína, crack | ○ | ○ | ○ |
| Anfetaminas ou êxtase | ○ | ○ | ○ |
| Inalantes | ○ | ○ | ○ |
| Hipnóticos/sedativos | ○ | ○ | ○ |
| Alucinógenos | ○ | ○ | ○ |
| Opioides | ○ | ○ | ○ |
| Outras | ○ | ○ | ○ |

Nosso questionário possui um último instrumento utilizado para avaliar níveis de estresse, ansiedade e depressão, cujo preenchimento ajudaria a compreender melhor suas respostas.

Deseja responder o questionário sobre estresse, ansiedade e depressão?

○ Sim
○ Não
(Obs: o preenchimento deste questionário é opcional)

**Questionnaire S4**. DASS-21 questionnaire (validated in Portuguese) (25).



**Por favor, leia cada afirmativa e marque aquela que indica o quanto a afirmativa aconteceu com você NA ÚLTIMA SEMANA. Não há respostas certas ou erradas. Não gaste muito tempo em nenhuma das afirmativas:**

| | Não aconteceu comigo nessa semana | Aconteceu comigo algumas vezes na semana | Aconteceu comigo em boa parte da semana | Aconteceu comigo na maior parte do tempo da semana |
|---|---|---|---|---|
| 1. Tive dificuldade em acalmar-me. | ○ | ○ | ○ | ○ |
| 2. Estava consciente de que minha boca estava seca. | ○ | ○ | ○ | ○ |
| 3. Parecia não conseguir ter nenhum sentimento positivo. | ○ | ○ | ○ | ○ |
| 4. Senti dificuldade em respirar (ex. respiração excessivamente rápida, falta de ar, na ausência de esforço físico). | ○ | ○ | ○ | ○ |
| 5. Tive dificuldade em tomar iniciativa para fazer as coisas. | ○ | ○ | ○ | ○ |
| 6. Tive a tendência de reagir de forma exagerada a situações. | ○ | ○ | ○ | ○ |
| 7. Senti tremores (ex.: nas mãos). | ○ | ○ | ○ | ○ |
| 8. Senti que estava geralmente muito nervoso. | ○ | ○ | ○ | ○ |
| 9. Preocupei-me com situações em que eu pudesse entrar em pânico e parecesse ridículo(a). | ○ | ○ | ○ | ○ |
| 10. Senti que não tinha nada a esperar do futuro. | ○ | ○ | ○ | ○ |
| 11. Senti que estava agitado. | ○ | ○ | ○ | ○ |
| 12. Tive dificuldade em relaxar. | ○ | ○ | ○ | ○ |
| 13. Senti-me desanimado e deprimido. | ○ | ○ | ○ | ○ |
| 14. Fui intolerante com as coisas que me impediam de continuar o que eu estava fazendo. | ○ | ○ | ○ | ○ |
| 15. Senti que ia entrar em pânico. | ○ | ○ | ○ | ○ |
| 16. Não consegui me entusiasmar com nada. | ○ | ○ | ○ | ○ |
| 17. Senti que não tinha muito valor como pessoa. | ○ | ○ | ○ | ○ |
| 18. Senti que estava sensível. | ○ | ○ | ○ | ○ |

| | | | | |
|---|---|---|---|---|
| 19. Eu estava consciente do funcionamento / batimento do meu coração na ausência de esforço físico (ex.: sensação de aumento da frequência cardíaca, disritmia cardíaca). | ○ | ○ | ○ | ○ |
| 20. Senti-me assustado sem ter uma boa razão. | ○ | ○ | ○ | ○ |
| 21. Senti que a vida estava sem sentido. | ○ | ○ | ○ | ○ |
